# Impact of Introduction of Arbuscular Mycorrhizal Fungi on the Root Microbial Community in Agricultural Fields

**DOI:** 10.1264/jsme2.ME18109

**Published:** 2018-12-22

**Authors:** Turgut Yigit Akyol, Rieko Niwa, Hideki Hirakawa, Hayato Maruyama, Takumi Sato, Takae Suzuki, Ayako Fukunaga, Takashi Sato, Shigenobu Yoshida, Keitaro Tawaraya, Masanori Saito, Tatsuhiro Ezawa, Shusei Sato

**Affiliations:** 1 Graduate School of Life Sciences, Tohoku University Sendai 980–8577 Japan; 2 Central Region Agricultural Research Center, National Agriculture and Food Research Organization (NARO) 2–1–18 Kannondai, Tsukuba 305–8666 Japan; 3 Kazusa DNA Research Institute Kisarazu 292–0818 Japan; 4 Graduate School of Agriculture, Hokkaido University Sapporo 060–8589 Japan; 5 Faculty of Agriculture, Yamagata University Tsuruoka 997–8555 Japan; 6 Field Science Center, Graduate School of Agriculture, Tohoku University Osaki 989–6711 Japan; 7 Western Region Agricultural Research Center, NARO Ayabe 623–0035 Japan; 8 Faculty of Bioresource Sciences, Akita Prefectural University Akita 010–0195 Japan; 9 Department of Innovation Research, Japan Science and Technology Agency Tokyo, 102–0076 Japan

**Keywords:** root microbiome, high-throughput community analysis, network analysis, microbiome manipulation, sustainable agriculture

## Abstract

Arbuscular mycorrhizal (AM) fungi are important members of the root microbiome and may be used as biofertilizers for sustainable agriculture. To elucidate the impact of AM fungal inoculation on indigenous root microbial communities, we used high-throughput sequencing and an analytical pipeline providing fixed operational taxonomic units (OTUs) as an output to investigate the bacterial and fungal communities of roots treated with a commercial AM fungal inoculum in six agricultural fields. AM fungal inoculation significantly influenced the root microbial community structure in all fields. Inoculation changed the abundance of indigenous AM fungi and other fungal members in a field-dependent manner. Inoculation consistently enriched several bacterial OTUs by changing the abundance of indigenous bacteria and introducing new bacteria. Some inoculum-associated bacteria closely interacted with the introduced AM fungi, some of which belonged to the genera *Burkholderia*, *Cellulomonas*, *Microbacterium*, *Sphingomonas*, and *Streptomyces* and may be candidate mycorrhizospheric bacteria that contribute to the establishment and/or function of the introduced AM fungi. Inoculated AM fungi also co-occurred with several indigenous bacteria with putative beneficial traits, suggesting that inoculated AM fungi may recruit specific taxa to confer better plant performance. The bacterial families *Methylobacteriaceae*, *Acetobacteraceae*, *Armatimonadaceae*, and *Alicyclobacillaceae* were consistently reduced by the inoculation, possibly due to changes in the host plant status caused by the inoculum. To the best of our knowledge, this is the first large-scale study to investigate interactions between AM fungal inoculation and indigenous root microbial communities in agricultural fields.

The root microbiome is vital for plant health and productivity (9, 76). Arbuscular mycorrhizal (AM) fungi are important members of this microbiome due to their ability to enhance plant nutrient uptake, particularly that of phosphorus (P) (68). Although P is crucial in plant nutrition, only 0.1% of total soil P is available to plants; therefore, P is a major limiting factor for plant growth (65). To overcome this issue, chemical P fertilizers have been used in agricultural systems. However, P fertilizers have negative impacts on the environment and are not suitable for sustainable agricultural production (65, 66). Since most globally important food crops naturally form AM symbiosis (67), sustainable P nutrition in agriculture may be maintained by manipulating the root microbiome through the introduction of AM fungi to increase the exploitation of soil P resources (10, 16, 54).

Bacteria appear to represent the third component of AM symbiosis because the establishment and function of AM fungi may be influenced by bacteria associated with the mycorrhizosphere (13, 25, 33). Previous studies reported the bacteria associated with the spores and hyphae of AM fungi (2, 6, 7, 56, 60, 63). These mycorrhizospheric bacteria, along with bacteria living in the endosphere, root nodules, and rhizosphere, may promote spore germination, hyphal growth, and AM fungal colonization (3, 7), and, thus, are known as mycorrhiza helper bacteria (MHB) (25). Furthermore, synergistic interactions between plant-growth-promoting rhizobacteria (PGPR) and AM fungi may improve plant yield (5, 45). For example, phosphate-solubilizing bacteria (PSB) and AM fungi can interact synergistically because PSB can increase the amount of available P from rock phosphate (5), resulting in increased plant P uptake and growth (26, 49).

The hyphae and spores of different AM fungal species associate with diverse bacteria (2, 56, 63). AM fungi have also been shown to stimulate or inhibit the growth of particular microbes (43). Consistent with these findings, shifts in the root microbiome following AM fungal inoculation have been reported. For example, a study that employed denaturing gradient gel electrophoresis revealed that AM fungal inoculation changed the root-associated bacterial and fungal communities of mesquite grown in a greenhouse with desert mine tailings (70). A more recent study using pyrosequencing demonstrated that AM fungal inoculation altered the root-associated bacterial communities of shrub species grown in degraded soil (55). Furthermore, by using Illumina MiSeq platform, it was revealed that *Deschampsia flexuosa* grown in a greenhouse with or without AM fungal inoculation had different bacterial community compositions in the leaves, but not in the roots, whereas the leaf- and root-associated fungal compositions of inoculated and non-inoculated plants were similar (50). However, there is no study that tracked the introduced AM fungi along with the bacteria that introduced together with the inoculum. Furthermore, the consistent effects of AM fungal inoculation on root bacterial communities in a broad range of agricultural fields have yet to be demonstrated.

High-throughput sequencing platforms are a powerful tool for simultaneously analyzing the dynamics of introduced AM fungi and bacteria in indigenous communities. Recently, we have developed a new analytical pipeline to provide taxonomic assignment of AM fungi for massive sequence reads generated by, e.g. MiSeq, via a web interface. (http://amfungi.kazusa.or.jp) (48). To apply this pipeline to bacteria, we constructed a reference data set of 16S rDNA sequences that fit the pipeline, which provides an ideal platform for a parallel analysis of fungal and bacterial communities. By using this tool, we investigated the root bacterial communities of Welsh onion (*Allium fistulosum* L.) to clarify the impact of AM fungal inoculation in a wide range of agricultural fields.

## Materials and Methods

### Fungal inoculum

The AM fungal inoculum used was *Glomus sp*. strain R-10 (hereafter R-10), purchased from Idemitsu Kosan, Tokyo, Japan. R-10 inoculum consists of spores, extraradical hyphae, and infected roots with a crystalline-silica carrier. Control plants were treated with a mock inoculum, *i.e*. a carrier that was free of propagule. Sequencing of R-10 inoculum showed that it included 21 AM fungal operational taxonomic units (OTUs), all likely from the *Rhizophagus* genus (48) (Table S1). These AM fungal OTUs were tracked in field studies. The use of this fungal inoculum was previously shown to have a positive effect on the yield of Welsh onion (71).

### Field trials and sample collection

All samples were collected from six different fields located across Japan in 2016 (Fig. S1). The indigenous bacterial and fungal community structures of the fields significantly different from each other (Fig. S2), and the fields had different characteristics and cultivation histories (Table S2). In Osaki, the three fields had different concentrations of available soil P due to previous fertilization: low P (OS1), medium P (OS2), and high P (OS3). The previous crop in OS1 and OS2 was Welsh onion, while it was potato in OS3. In Tsuruoka (TRO), the previous crop was also Welsh onion. Ayabe (AYB) and Tsugaru (TGR) fields were bare fallowed in the previous season. P fertilizer was applied at two (P0 and P100) or three levels (P0, P50, and P100) at each field area, as reported by Tawaraya *et al*. (71): 0 (P0) and 100 (P100) kg P_2_O_5_/10 a at OS1 and OS2 and 0 (P0), 50 (P50), and 100 (P100) kg P_2_O_5_/10 a at AYB, OS3, TGR, and TRO. Welsh onion cv. Motokura was used for all field experiments. Welsh onion seedlings were grown with the mock or R-10 inoculum in plug trays for 50 d under greenhouse conditions and were then transferred to the fields, as described previously (71). The treatments were arranged in a randomized block design, except for TRO, with a completely randomized design being applied to minimize the effects of available P concentrations in each plot.

Soil samples were collected from each replicated block (*n*=4) prior to the application of fertilizer, freeze-dried, and stored at −30°C for DNA extraction and sequencing (at TRO, samples were collected from pseudo-replicated blocks, *n*=4). Roots were sampled at two plant growth stages, approximately 1 and 2 months after transplanting (hereafter 1 and 2 MAT). In total, 256 samples were collected (3 fertilizer levels×2 inoculation types [mock and R-10]×2 plant growth stages×4 replicates at AYB, OS3, TGR, and TRO; 2 fertilizer levels×2 inoculation types×2 plant growth stages×4 replicates at OS1 and OS2). Root samples were collected as described previously (48). Briefly, roots and root-zone soils (20×20 cm square, depth of 25 cm) were collected from four plants in each plot and combined. The roots were gently washed with tap water, and approximately 1 g of subsamples (lateral roots attached to the tap root) were collected in the middle of the tap root, cut into 1-cm segments, randomized in water, blotted on a paper towel, freeze-dried, and stored at −30°C for DNA extraction. In measurements of the dry weight and P concentration of Welsh onion, another four plants were harvested in each plot, combined, dried at 80°C for 72 h, and ground with a mill. P concentrations were estimated as described by Niwa *et al*. (48).

### DNA extraction, PCR amplification, sequencing, and bioinformatics

The targeted metagenomic profiling of samples was performed by sequencing the V3–V4 region of the 16S rDNA gene for bacteria and the D2 region of the 28S rDNA gene for fungi. The V3–V4 region was amplified according to the Illumina MiSeq 16S Metagenomic Sequencing Library Preparation protocol (http://support.illumina.com/downloads/16s_metagenomic_sequencing_library_preparation.html). The D2 region in LSU rDNA was amplified with the forward primer FLd3-1 (48) and reverse primer FLR2 (72). Sample preparation, DNA extraction, and gene amplification were performed as described previously (48). We conducted 300-bp paired-end sequencing on the Illumina MiSeq Instrument using the 600-cycle v3 Sequencing Kit.

Regarding fungal reads, we used the approach that we described previously, which includes quality filtering, the merging of paired-end reads, OTU picking, and assigning taxonomies (48). The fungal database implemented in this pipeline was based on previously published AM fungal sequences and 28S rDNA sequences obtained from the Ribosomal Database Project (RDP) version 11.4 (19).

We developed a similar approach for 16S rDNA sequences. A bacterial database was created based on 16S rDNA sequences in RDP version 11.4 (19). 16S rDNA sequences ranging between 1330 and 1500 nt were clustered using CD-HIT-EST with identity≥97%, E-value≤1.0e–100, and alignment length≥1200 nt (37). The quality trimming of reads was conducted by PRINSEQ version 0.20.4 (61), and overlap fragments were constructed using COPE version 1.1.13 (39) with minimum and maximum overlap lengths of 10 and 300 nt, respectively. Overlap fragments were subjected to BLASTN searches with E-value≤1.0e–100, alignment length>330 nt, and identity≥97% against the 16S rDNA sequence data set that we created.

To validate the new pipeline for bacterial reads, we also employed UPARSE (USEARCH version 10.0.240) on randomly selected samples (22). Nucleotides with a quality value lower than 30 in the 3′ terminal were trimmed. The threshold for the maximum number of mismatches in the alignment was 7. We set the maximum expected error threshold to 1.0 for quality filtering. We excluded sequences if they were shorter or longer than 300 and 580 nt, respectively. High quality reads were clustered at 97% identity to identify OTUs. Taxonomies were assigned to each OTU using the USEARCH algorithm at 90% identity with the GREENGENES database (21).

### Statistical analysis

All statistical analyses were performed in R version 3.3.3 (51). Bulk soil samples were compared using *pairwise.perm.manova* from the package *RVAideMemoire* (Herve, M. 2018. RVAideMemoire: Testing and Plotting Procedures for Biostatistics). To validate the new pipeline, we performed Mantel test and Procrustes analysis with the functions *mantel* and *protest* in the package *vegan*, respectively (Oksanen, J., F.G. Blanchet, R. Kindt, *et al*. 2013. Vegan: community ecology package). The dry weight and P content response to R-10 inoculation were calculated with log_2_(*X*_R_/*X*_M_), where *X*_R_ is the dry weight or P content in R-10-inoculated plants and *X*_M_ is the mean of the respective parameters in mock-inoculated samples. In the beta diversity analysis (constrained analysis of principal coordinates [CAP] and permutational analysis of variance [PERMANOVA]), bacterial and fungal OTU tables were filtered to exclude OTUs with low abundance (*i.e*. occurred at less than 0.05 and 0.01% in all samples in the bacterial and fungal OTU tables, respectively). Filtered data were rarefied to the minimum sequencing depth of the respective OTU table. CAP was performed based on Bray–Curtis dissimilarities using the package *phyloseq* (44). The significance of CAP was evaluated using the *permutest* function in the *vegan* package with 5,000 permutations (Vegan: community ecology package). The drivers of the bacterial and fungal community structures were assessed with PERMANOVA using the *adonis* function in the R package *vegan* with 5,000 permutations (Vegan: community ecology package). To identify the OTUs responsible for the discrimination between mock- and R-10-inoculated samples, we performed indicator species analysis using the *multipatt* function in the package *indicspecies* (15). *P* values were estimated with 5,000 permutations and corrected for multiple testing using the Benjamini–Hochberg method (8).

We performed a network analysis with a similar approach to that described by Hartman *et al*. (27). Briefly, we normalized OTU counts using the *varianceStabilizingTransformation* function in the *DESeq2* package (40). Bacterial and fungal OTU tables were then combined and Spearman’s rank correlations between each OTU were calculated. The positive and statistically significant correlations (ρ>0.6 and *P*<0.001) were visualized with a Fruchterman–Reingold layout with 5,000 permutations in the package *igraph* (20). The clusters in the network were detected with the *igraph* function *cluster_louvain* (12).

## Results

### Validation of the analytical pipeline

By clustering 1,290,478 bacterial 16S rDNA sequences, 307,998 sequences including 7,292 type strains were obtained and defined as reference sequences for the OTU assignment in the new pipeline (the reference sequence data set is available via the web interface; http://amfungi.kazusa.or.jp). To evaluate the performance of the new pipeline, we initially compared it with UPARSE to confirm its applicability. We randomly selected six samples from each field (three samples at each plant growth stage) and applied both of the pipelines to these samples. UPARSE detected 5,871 OTUs. Among these were 157 chloroplast, 78 mitochondrial, and 1,519 unassigned sequences. After removing these sequences, there were 1,326 OTUs that occurred in only one sample, which may have arisen due to a sequencing error. Therefore, we discarded the singletons and 2,791 OTUs remained. The new pipeline found 5,945 OTUs in the 36 randomly selected samples, of which 6 were chloroplast and 1,835 were singleton sequences. After excluding these reads, we obtained 4,014 OTUs. Hence, we were able to recover more OTUs using the new pipeline.

We also investigated whether these pipelines produced similar patterns among the samples. To achieve this, we performed principal coordinates analysis (PCoA), Procrustes analysis, and Mantel test at both the OTU and family level (Fig. S3). Procrustes analyses were conducted on the first six coordinates and revealed very low errors at both the OTU and family level (*M*^2^=0.067 and *M*^2^=0.045, respectively), and Mantel test produced similar results (*r*=0.97 and *r*=0.98, respectively). Therefore, we concluded that it was valid to continue with the new pipeline.

We defined three outlier samples (one from OS3 and two from TGR) by preliminary PCoAs and excluded these from further analyses. Regarding the remaining 253 samples, bacterial community profiling yielded a total of 5,791,128 high-quality sequences ranging between 3,309 and 86,755 with a median of 18,895. Fungal community profiling yielded a total of 5,710,514 high-quality sequences ranging between 5,284 and 65,983 with a median of 21,371. We identified 11,229 bacterial OTUs, 16 of which were assigned to chloroplast and were, thus, discarded. The number of fungal OTUs was 976, of which 209 were AM fungal OTUs.

### Background bacterial communities of R-10 inoculum

As a first step, we analyzed the root bacterial communities associated with the inocula using Welsh onion seedlings at the nursery stage (*i.e*. those grown in the greenhouse). PERMANOVA indicated that the compositions of the sterilized and intact mock inoculum treatments were not significantly different (*P*=0.6745). The community in the intact R-10 inoculum treatment was significantly different from the sterilized R-10 inoculum and mock inoculum treatments (PERMANOVA, *P*=0.0002 for both tests). We found the enrichment of 59 and 50 OTUs in the intact R-10 inoculum treatment with respect to the sterilized R-10 inoculum and mock inoculum treatments, respectively, in which 44 OTUs were commonly enriched (Table S3). Therefore, we defined these 44 OTUs as R-10 inoculum-associated bacteria.

### Factors driving the root microbial community structure

**Fig. 1:**
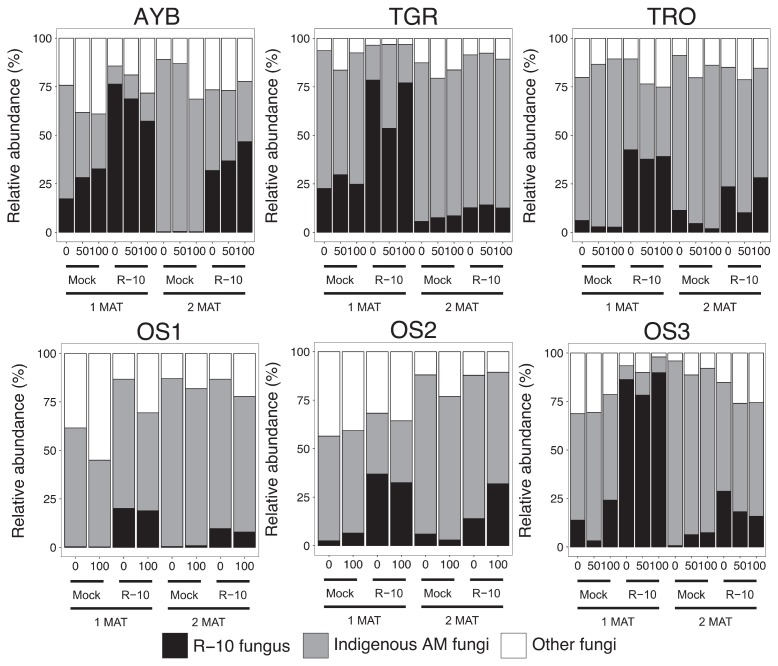
Stacked bar plots depicting the average relative abundance (%) of R-10-type OTUs, indigenous AM fungal OTUs, and other indigenous fungal OTUs in mock- (control) and R-10-inoculated samples at each P fertilizer level (*n*=4).

R-10 inoculum consists of 21 AM fungal OTUs that were employed as a tracking marker in the fields. However, these OTUs are also common in indigenous fungal communities (29, 48), and, thus, colonization of the inoculum fungus was only inferred based on relative increases in the abundance of R-10-type OTUs from those in mock-inoculated (control) plants. The cumulative abundance of R-10-type OTUs was significantly higher in inoculated plants than in mock plants in all fields at 1 MAT (Fig. 1). In 2 MAT samples, the total abundance of R-10-type OTUs decreased, and the difference between mock- and R-10-inoculated plants was slightly ambiguous, except for that in AYB.

**Fig. 2:**
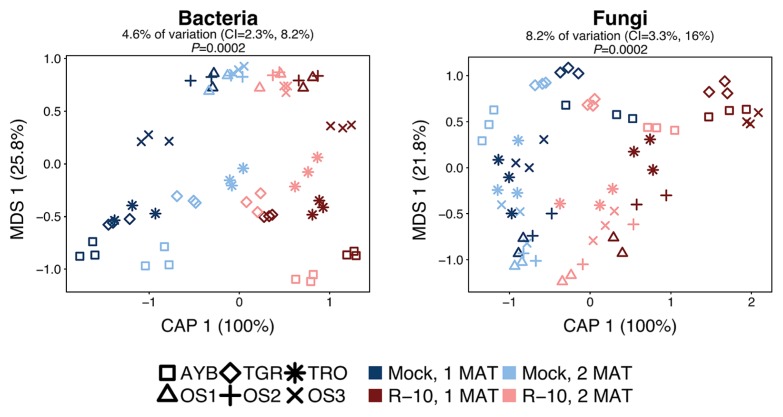
Effect of R-10 inoculum on bacterial and fungal communities associated with Welsh onion roots. CAP with Bray-Curtis dissimilarities were constrained by the factor “R-10 inoculum”. CAP was applied on the average read abundance in mock- and R-10-inoculated samples at each P fertilizer level (*n*=4). The variation explained by the constrained factor with the 95% confidence interval (CI) and significance was given above each plot. The remaining unconstrained ordination was subjected to multi-dimensional scaling (MDS), the first MDS axis (MDS 1) is shown. The variation explained (%) by each axis is given in parentheses.

We then examined whether the inoculation shifted the bacterial and fungal assemblages associated with Welsh onion roots. We performed PERMANOVA on Bray–Curtis dissimilarities to reveal the drivers of the microbial community structure (Table 1). The introduction of R-10 fungus significantly altered root bacterial communities (explaining 4.5% of the variance). Field and plant growth stage were also determinants of bacterial communities (42.4 and 5.1% of the variation, respectively). R-10 inoculum also significantly influenced the fungal composition (explaining 8.2% of the variation), and field and plant growth stage significantly changed the fungal community structure (36 and 5% of the variation, respectively). The impact of P fertilizer on bacterial and fungal root communities was marginal (0.5% of the variation, *P*=0.0174 for bacteria; *P*=0.5095 for fungi). The interaction of plant growth stage×R-10 inoculum significantly affected the structures of both the bacterial and fungal root communities (1.2 and 1.3% of the variation, respectively). The results of CAP indicated that the effects of R-10 inoculum on microbial communities were more distinct at 1 MAT than at 2 MAT (Fig. 2). Therefore, we used the data obtained at 1 MAT in subsequent analyses.

### Taxa responsive to R-10 fungus

To identify the microbial taxa responsive to R-10 inoculum (*i.e*. enriched or decreased by R-10 inoculum), we performed indicator species analysis separately on each field at the family and OTU level. To assess the general effect of R-10 inoculum, we defined the microbes that were consistently responsive to R-10 inoculum (*i.e*. in five or more fields) as CER or CDR (consistently enriched/decreased by R-10 inoculum).

The indicator species analysis of the fungal communities revealed that R-10 inoculum not only changed the indigenous AM fungi, but also had an impact on other fungal taxa (Fig. S4, Table S4 and S5). There were 83 OTUs (23 enriched and 60 decreased) that responded to R-10 inoculum, and 20 fungal families were decreased by R-10 inoculum. However, there was no CDR fungal families or OTUs, and the only CER OTUs were R-10-type OTUs (019_Rhz_AB640747 and 392_Rhz_LC191629), suggesting that R-10 inoculum affected indigenous AM fungi and other fungal members in a field-dependent manner.

**Fig. 3:**
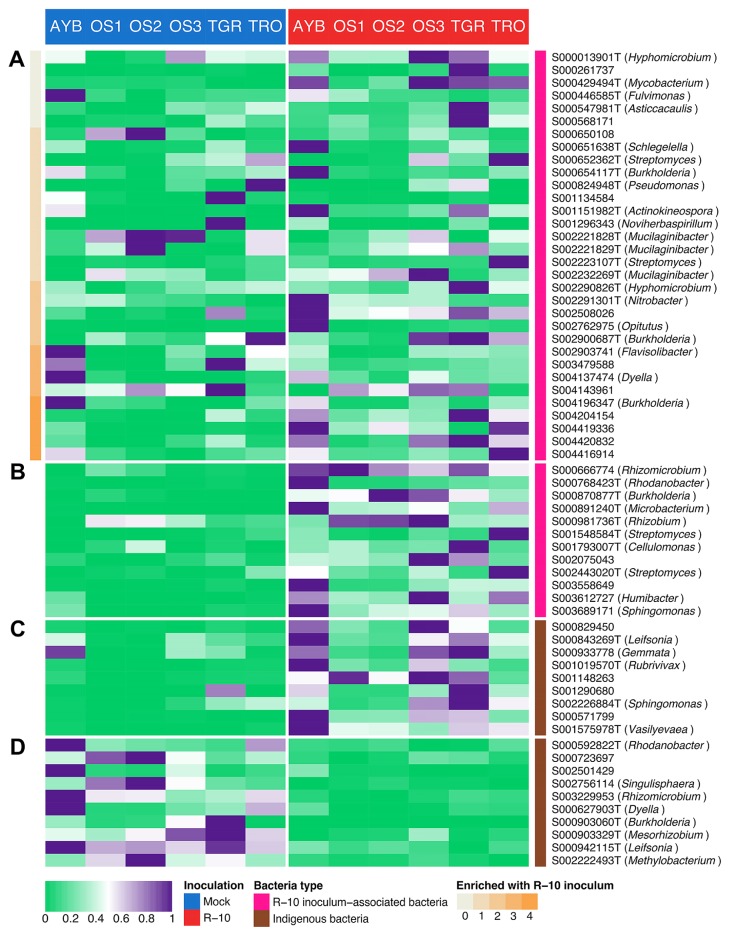
Heatmap showing the average relative abundance (%) of **(A)** non-CER R-10 inoculum-associated bacterial OTUs, **(B)** CER R-10 inoculum-associated bacterial OTUs, **(C)** indigenous CER bacterial OTUs, and **(D)** indigenous CDR bacterial OTUs in 1 MAT samples. Average values were calculated by combining samples from different P fertilizer levels (*n*=12 for AYB, OS3, TGR, and TRO; *n*=8 for OS1 and OS2). The relative abundance (%) of each OTU was scaled to a 0–1 interval. Non-CER R-10 inoculum-associated bacterial OTUs are ordered by the number of the fields in which they were enriched by R-10 inoculum (indicated in orange at the left of the heatmap). The types of bacteria are also depicted (R-10 inoculum-associated bacteria and indigenous bacteria).

In bacterial communities, we found that 52 families (19 enriched and 33 decreased) and 522 OTUs (211 enriched and 311 decreased) were responsive to R-10 inoculum in 1 MAT samples. In enriched taxa, we did not detect a CER family from a firmly assigned family (the only CER family was assigned to “Burkholderiales_incertae_sedis”, Fig. S5). At the OTU level, we detected 21 CER bacterial OTUs (Fig. 3B and C, Table S6). Among 44 R-10 inoculum-associated bacterial OTUs, 12 were identified as CER, suggesting that a portion of inoculum-associated bacteria accumulated in the roots regardless of the environmental conditions. These OTUs belonged to the genera *Burkholderia*, *Cellulomonas*, *Microbacterium*, *Rhizobium*, *Rhodanobacter*, *Sphingomonas*, and *Streptomyces*. In addition, 9 OTUs that were not among inoculum-associated bacterial OTUs were also identified as CER, suggesting that R-10 inoculum enriched some indigenous bacterial species. Some of these indigenous OTUs were assigned to the genera *Gemmata*, *Leifsonia*, *Rubrivivax*, *Sphingomonas*, and *Vasilyevaea*.

**Fig. 4:**
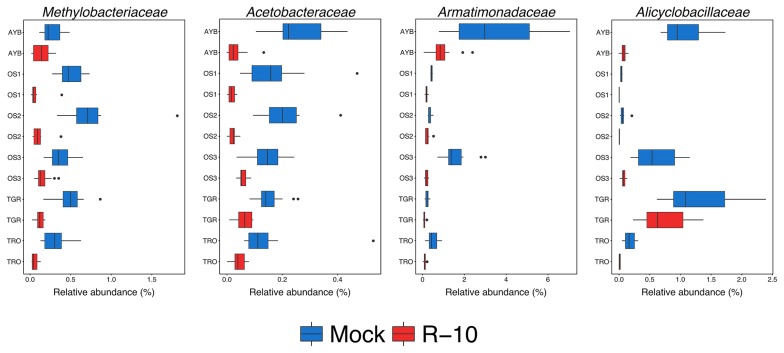
Boxplots depicting the relative abundance (%) of bacterial CDR families. The vertical bars within boxes represent medians. Medians were calculated by combining samples from different P fertilizer levels (*n*=12 for AYB, OS3, TGR, and TRO; *n*=8 for OS1 and OS2). Boxplot whiskers extend the interquartile range 1.5-fold from the upper and lower quartiles. Outlier values are shown as individual points.

In decreased taxa, *Methylobacteriaceae*, *Acetobacteraceae*, *Armatimonadaceae*, and *Alicyclobacillaceae* were identified as CDR bacterial families (Fig. 4 and S5). We also detected 10 CDR at the OTU level (Fig. 3D, Table S6). Among them, S002222493T (*Methylobacteriaceae*), S000723697 (*Acetobacteraceae*), and S002501429 (*Armatimonadaceae*) overlapped with the CDR families. However, these CDR OTUs were not the only explanation for CDR families because non-CDR OTUs (decreased in less than 5 fields) also contributed to consistent family-level reductions: S000925429T (*Methylobacteriaceae*); S003435437, S001093906T, and S000650108 (*Acetobacteraceae*); S000840854 (*Armatimonadaceae*); and S000627911T and S000842399T (*Alicyclobacillaceae*).

### Co-occurrence patterns in the root microbiome

**Fig. 5:**
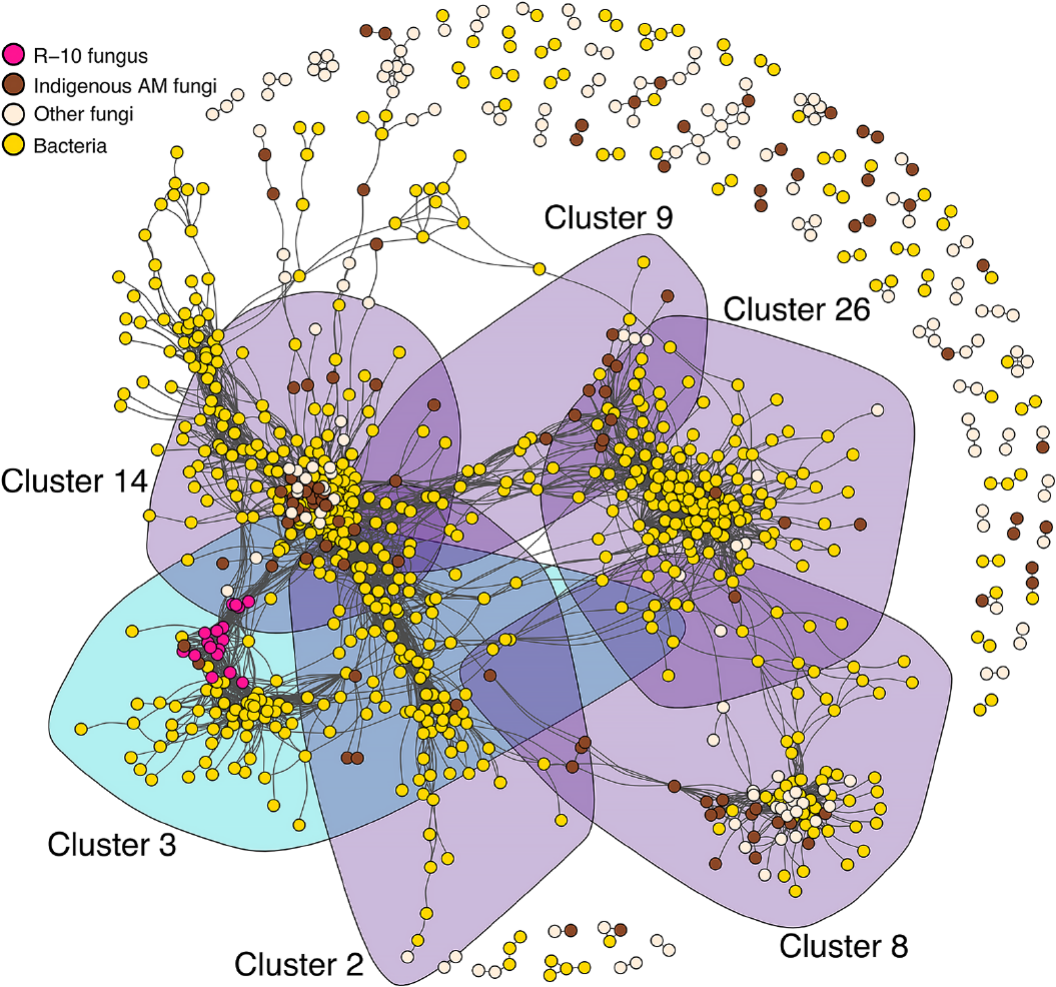
Network analysis of root-associated bacterial and fungal OTUs in 1 MAT samples. The co-occurrence network shows positive correlations (indicated with gray lines) between each OTU. The clusters are shaded with respect to the AM fungus type, *i.e*. the turquoise area represents R-10 fungus cluster, whereas the purple areas represent indigenous AM fungal clusters.

The effects of R-10 inoculum on the root microbiome may be direct or indirect (via changes in the plant status); therefore, we used a network analysis to assess the effects of R-10 inoculum on responsive taxa. The network constructed with bacterial and fungal OTUs from 1 MAT samples revealed that all R-10-type OTUs were clustered in cluster 3 (Fig. 5). Among the 12 R-10 inoculum-associated bacterial CER OTUs, 11 (except for S000981736T, which was assigned to *Rhizobium*) were placed in cluster 3, indicating direct interactions between R-10 fungus and these bacteria (Table S7). All 9 indigenous bacterial CER OTUs were placed in cluster 3, suggesting that they directly associated with R-10 fungus (Table S7). In addition to these CER OTUs, 34 indigenous bacterial OTUs co-occurred in cluster 3, including OTUs from the genera *Microbacterium* and *Sphingomonas*, which overlapped with R-10 inoculum-associated CER OTUs (Table S7).

We also found five main indigenous AM fungal clusters with four or more AM fungal OTUs (clusters 2, 8, 9, 14, and 26; Fig. 5). Among these, the AM fungi of cluster 26 negatively correlated with R-10 fungus (Fig. S6A), suggesting niche competition as described previously (48). These indigenous AM fungal OTUs were from the genera *Gigaspora* (163_Gig_AB665514, 164_Gig_GQ229230, and 165_Gig_FN547559) and *Acaulospora* (186_Aca_AF378437). There were also 15 bacterial OTUs in cluster 26 that negatively interacted with R-10 fungus (ρ<−0.6 and *P*<0.001, Fig. S6B). These 15 bacterial OTUs were assigned to the genera *Arthrobacter*, *Bradyrhizobium*, *Pseudomonas*, and *Ralstonia*, none of which overlapped with the 10 bacterial CDR OTUs (Table S8). Additionally, except for S000840854 (*Armatimonadaceae*), none of the non-CDR OTUs contributing to the aforementioned consistent family-level reductions negatively correlated with R-10 fungus.

## Discussion

In the present study, we assessed the impact of AM fungal inoculation on root microbial communities by elucidating the interactive networks of introduced and indigenous microorganisms using a newly developed molecular tool. We, for the first time, demonstrated that bacteria that associate with an introduced AM fungus, at least some of them, are capable of persisting across environments, and the introduced AM fungus may consistently affect some indigenous bacterial taxa in a wide range of agricultural fields, implying that the influence of introducing exotic AM fungi on root bacterial communities is unexpectedly strong.

We identified the drivers of bacterial and fungal community structures (Table 1). Field was the most influential factor affecting the root microbial community structure. Root-associated microbes mainly originate from soil; therefore, variations in the indigenous microbial communities of bulk soil (Fig. S2) reflected those in environmental conditions, soil types, agricultural practices, and previous cultivars among fields, which were the main determinants of the microbial community structure, as noted previously (23, 64). Plant growth also had a significant effect on root microbial assemblages, as reported previously (18, 38, 42, 57). Time-dependent shifts in root-associated microbial compositions may reflect temporal changes in root exudation as carbohydrate partitioning and exudation changes with plant age and the distinct stages of plant growth (18, 58, 75). Despite the large variations observed in indigenous microbial communities among fields, R-10 inoculum altered root-associated bacterial and fungal communities in all fields (Fig. 2, Table 1). The introduction of AM fungi may also introduce their associated bacteria, and, because different AM fungal species are associated with diverse bacteria (2, 56, 63), AM fungal inoculation may lead to changes in root bacterial composition. Furthermore, AM fungi may stimulate or inhibit the growth of particular microbes (43) by providing carbon compounds derived from host assimilates to mycorrhizospheric bacteria via mycelia, competing for nutrients with bacteria, and exuding inhibitory or stimulatory compounds (2, 24, 33, 74). Besides these direct effects of AM fungal inoculation, introduced AM fungi may also have an indirect effect on the root microbiome via changing the root exudation patterns, caused by improved plant status (69). For example, a shift in the root bacterial community following the AM fungal inoculation was previously shown to be related to a change in shoot P uptake (55).

We performed indicator species analysis to identify which taxa explained the differences between mock- and R-10-inoculated community structures. Among the 21 bacterial CER OTUs, 12 were inoculum-associated bacteria, of which 11 OTUs were clustered with R-10-type OTUs in the network analysis, suggesting that they were closely associated with R-10-type OTUs (Fig. 3B and 5; Table S6 and S7). Hence, these bacterial OTUs may represent the mycorrhizospheric bacteria of R-10 fungus, which may function as MHB, PSB, or both to promote mycorrhizal establishment, function, or both (6, 7). Although it is difficult to infer the ecological function of a microbe based on its taxonomic assignment (36), the robust associations of introduced AM fungi and bacteria independent of soil characteristics imply that these bacteria are not solely contaminants of the inoculum, but instead may be bacteria protected by the introduced fungi, suggesting that fungi benefit from them. For example, some of the 11 inoculum-associated bacterial CER OTUs with direct interactions with R-10-type OTUs in the network analysis were assigned to the genera *Burkholderia*, *Cellulomonas*, *Microbacterium*, *Sphingomonas*, and *Streptomyces*, which include taxa with MHB and/or PSB traits. *Burkholderia* and *Microbacterium* are among the most significant PSB (11). *Streptomyces* species serve as MHB for both ectomycorrhizal and AM fungi (1, 7, 17, 53, 62), are abundant on the hyphal surfaces of AM fungi (60), promote plant growth (28), and protect against plant pathogens (14). *Sphingomonas* species act as both PSB (11, 47) and MHB (30). *Cellulomonas* species produce auxin (73) and colonize the hyphal surfaces of AM fungi (60). *Cellulomonas* species were also isolated from the mycelia of AM fungi (41). With these closely associated bacteria in the inoculum, R-10 fungus may perform well as a biofertilizer in Welsh onion cultivation (Fig. S7). Furthermore, 13 non-CER inoculum-associated bacterial OTUs (*i.e*. enriched in less than five fields) also co-occurred with R-10-type OTUs in the network analysis. Bacteria assigned to *Burkholderia*, *Mycobacterium*, and *Nitrobacter* were among the 13 OTUs. *Mycobacterium* produce auxin and thereby facilitate plant growth (73), and *Nitrobacter* species act as PSB (31, 65). Thus, these candidate mycorrhizospheric bacteria may play an important role in the positive effects of R-10 fungus on Welsh onion plants (Fig. S7).

A total of 43 indigenous bacteria were placed in cluster 3, including all 9 indigenous CER OTUs (Fig. 3C and 5; Table S6 and S7). Two of these CER OTUs were assigned to the genera *Leifsonia* and *Sphingomonas*, members of which act as PSB (11, 46, 47). Among the 34 non-CER indigenous bacteria co-occurring with R-10 fungus, five of the OTUs were assigned to *Bacillus*, *Caulobacter*, *Microbacterium* (two OTUs), and *Sphingomonas*. Along with the aforementioned putative positive functions of *Microbacterium* and *Sphingomonas* (11), *Caulobacter* was described as PGPR and *Bacillus* as PSB (11). Hence, R-10 inoculum may have a direct impact on the indigenous bacterial community, resulting in the recruitment of bacteria that exert positive effects on the plant and, thus, improve plant performance (Fig. S7).

In contrast to the CER in bacterial communities, CDR bacteria were detected at the family level (Fig. 4 and S5). We identified four CDR families in the bacterial community of 1 MAT samples (*Methylobacteriaceae*, *Acetobacteraceae*, *Armatimonadaceae*, and *Alicyclobacillaceae*). Specifically, *Methylobacteriaceae* and *Acetobacteraceae* were reduced by R-10 inoculum in all fields. *Methylobacteriaceae* members are methylotrophs that use single carbon compounds as energy sources (35), and actively participate in carbon cycling in soil (32, 34). In addition, some members of this family are non-symbiotic nitrogen-fixing bacteria (4). *Acetobacteraceae* members oxidize sugars and alcohols to produce organic acids as final products of their metabolism (52), and this family also includes non-symbiotic nitrogen-fixing bacteria (59). Therefore, the consistent changes observed in the abundance of these families following inoculation suggest that R-10 inoculum may change carbon and nitrogen metabolism in Welsh onion roots. These family-level changes may have been related to alterations in the plant status because the OTUs contributing to these changes were not clustered with any indigenous AM fungi in the network analysis or negatively correlated with R-10-type OTUs (except the non-CDR OTU S000849854 from *Armatimonadaceae*, Table S8). This result suggests that these family-level alterations were an indirect effect of R-10 inoculum. Therefore, we surmise that R-10 inoculum led to changes in the root microbiome both directly (for the enriched taxa) and indirectly (for the decreased taxa).

It is important to note that there may be more indigenous taxa consistently responsive to R-10 inoculation than we provided here. Although we confirmed the results of the indicator species analysis using DESeq2 (40) (data not shown), the within variations were large, particularly in fungal data; therefore, it is possible that we missed some CER and/or CDR taxa in our analyses. Furthermore, the CDR taxa that we found were constrained to the OTUs that were present in all fields; therefore, there may be some indigenous CDR OTUs that were not detected in the present study because they did not exist in some of the fields. Future studies with more fields and bigger sample sizes will help to elucidate AM fungal inoculation-indigenous microbiome interactions more clearly. Nevertheless, we consider the present results to provide valuable insights for further research.

In conclusion, we herein demonstrated the microorganisms responded to AM fungal inoculation regardless of environmental conditions. Furthermore, we identified the bacterial OTUs closely associated with the introduced AM fungi, which may have contributed to mycorrhizal establishment, function, or both, likely leading to improved performance in Welsh onion cultivation (Fig. S7). However, since we inferred the functions of bacteria based only on taxonomic assignments, further laboratory experiments are needed to verify our results. Collectively, our field and laboratory results will facilitate the development of better approaches for manipulating the root microbiome in order to increase the sustainability of agricultural systems.

## Supplementary Information



## Figures and Tables

**Table 1:** Drivers of bacterial and fungal beta diversities assessed by PERMANOVA based on Bray-Curtis dissimilarities. Only significant factors are displayed.

	Variation Explained (%)	*P* value
**Bacteria**		

Field	42.4	***
Plant growth stage	5.1	***
R-10 inoculum	4.5	***
P fertilizer	0.5	*
Field×Plant growth stage	8.8	***
Field×R-10 inoculum	4.6	***
Plant growth stage×R-10 inoculum	1.2	***
Field×Plant growth stage×R-10 inoculum	1.5	***

**Fungi**		

Field	36	***
Plant growth stage	5	***
R-10 inoculum	8.2	***
Field×Plant growth stage	10.6	***
Field×R-10 inoculum	3.7	***
Plant growth stage×R-10 inoculum	1.3	***
Field×Plant growth stage×R-10 inoculum	2.4	***

* *P*<0.05,

*** *P*<0.001
