# Supplementary material for: Impact of Introduction of Arbuscular Mycorrhizal Fungi on the Root Microbial Community in Agricultural Fields
Source: Microbes Environ. 2018 Dec 22;34(1):23–32. doi: 10.1264/jsme2.ME18109 (PMC6440726; doi:10.1264/jsme2.ME18109)
Supplement: Supplementary file 1 [file 34_23_s1.pdf]

SUPPLEMENTARY TABLES

Table S1. AM fungal OTUs found in the inoculum *Glomus* sp. strain R-10. The OTU codes were created by Niwa *et al.* (48).

**Code**
001_Rhz_AB369921
006_Rhz_FM865608
007_Rhz_AB369745
008_Rhz_AB640745
019_Rhz_AB640747
230_Rhz_JN937313
231_Rhz_FR750064
367_Rhz_LC191607
373_Rhz_LC191612
374_Rhz_LC191613
375_Rhz_LC191614
377_Rhz_LC191616
379_Rhz_LC191618
380_Rhz_LC191619
381_Rhz_LC191620
382_Rhz_LC191621
387_Rhz_LC191625
388_Rhz_LC191626
390_Rhz_LC191627
391_Rhz_LC191628
392_Rhz_LC191629

Table S2. Soil type and cultivation history of the fields used in the study.

Abbreviation	Location	Soil Type	Cultivation History		
			2013	2014	2015
AYB	Iden, Ayabe city, Kyoto prefecture	Brown lowland soil	Oat, sorghum	Oat, sorghum	Fallow
OS1	Naruko-onsen, Osaki city, Miyagi prefecture	Non-allophanic andosol	Soybean	Soybean	Welsh onion
OS2	Naruko-onsen, Osaki city, Miyagi prefecture	Non-allophanic andosol	Soybean	Soybean	Welsh onion
OS3	Naruko-onsen, Osaki city, Miyagi prefecture	Non-allophanic andosol	Fallow	Fallow	Potato
TGR	Dekishima, Tsugaru city, Aomori prefecture	Gray lowland soil	Fallow	Fallow	Fallow
TRO	Wakaba-cho, Tsuruoka city, Yamagata prefecture	Brown lowland soil	Welsh onion	Fallow	Welsh onion

Table S3. Bacterial OTUs associated with R-10 inoculum.

RDP Code	Phylum	Class	Order	Family	Genus	Cluster [a]	Count [b]
S000654117T	*Proteobacteria*	*Betaproteobacteria*	*Burkholderiales*	*Burkholderiaceae*	*Burkholderia*		
S000824948T	*Proteobacteria*	*Gammaproteobacteria*	*Pseudomonadales*	*Pseudomonadaceae*	*Pseudomonas*		
S001134584	"Candidatus Saccharibacteria"				"Saccharibacteria_genera_incertae_sedis"		
S001296343	*Proteobacteria*	*Betaproteobacteria*	*Burkholderiales*	*Oxalobacteraceae*	*Noviherbaspirillum*		
S004143961	*Proteobacteria*	*Deltaproteobacteria*					
S004204154	"Candidatus Saccharibacteria"				"Saccharibacteria_genera_incertae_sedis"		
S000652362T	*Actinobacteria*	*Actinobacteria*	*Actinomycetales*	*Streptomycetaceae*	*Streptomyces*	2	
S000650108	*Proteobacteria*	*Alphaproteobacteria*	*Rhodospirillales*	*Acetobacteraceae*		26	
S002221828T	*Bacteroidetes*	*Sphingobacteriia*	*Sphingobacteriales*	*Sphingobacteriaceae*	*Mucilaginibacter*	26	
S000568171	"Candidatus Saccharibacteria"				"Saccharibacteria_genera_incertae_sedis"		1
S002223107T	*Actinobacteria*	*Actinobacteria*	*Actinomycetales*	*Streptomycetaceae*	*Streptomyces*		1
S002290826T	*Proteobacteria*	*Alphaproteobacteria*	*Rhizobiales*	*Hyphomicrobiaceae*	*Hyphomicrobium*		1
S002900687T	*Proteobacteria*	*Betaproteobacteria*	*Burkholderiales*	*Burkholderiaceae*	*Burkholderia*		1
S000446585T	*Proteobacteria*	*Gammaproteobacteria*	*Xanthomonadales*	*Xanthomonadaceae*	*Fulvimonas*	3	1
S001151982T	*Actinobacteria*	*Actinobacteria*	*Actinomycetales*	*Pseudonocardiaceae*	*Actinokineospora*	3	1
S004196347	*Proteobacteria*	*Betaproteobacteria*	*Burkholderiales*	*Burkholderiaceae*	*Burkholderia*	3	1
S000261737	"Candidatus Saccharibacteria"				"Saccharibacteria_genera_incertae_sedis"	25	1
S002221829T	*Bacteroidetes*	*Sphingobacteriia*	*Sphingobacteriales*	*Sphingobacteriaceae*	*Mucilaginibacter*		2
S002903741	*Bacteroidetes*	*Sphingobacteriia*	*Sphingobacteriales*	*Chitinophagaceae*	*Flavisolibacter*		2
S000651638T	*Proteobacteria*	*Betaproteobacteria*	*Burkholderiales*	*Comamonadaceae*	*Schlegelella*	3	2

S002291301T	*Proteobacteria*	*Alphaproteobacteria*	*Rhizobiales*	*Bradyrhizobiaceae*	*Nitrobacter*	3	2
S002232269T	*Bacteroidetes*	*Sphingobacteriia*	*Sphingobacteriales*	*Sphingobacteriaceae*	*Mucilaginibacter*	26	2
S003479588	"Candidatus Saccharibacteria"				"Saccharibacteria_genera_incertae_sedis"	27	2
S000547981T	*Proteobacteria*	*Alphaproteobacteria*	*Caulobacterales*	*Caulobacteraceae*	*Asticcacaulis*	2	3
S000013901T	*Proteobacteria*	*Alphaproteobacteria*	*Rhizobiales*	*Hyphomicrobiaceae*	*Hyphomicrobium*	3	3
S002762975	*Verrucomicrobia*	*Opitutae*	*Opitutales*	*Opitutaceae*	*Opitutus*	3	3
S004416914	"Candidatus Saccharibacteria"				"Saccharibacteria_genera_incertae_sedis"	3	3
S000429494T	*Actinobacteria*	*Actinobacteria*	*Actinomycetales*	*Mycobacteriaceae*	*Mycobacterium*	3	4
S002508026	*Chloroflexi*					3	4
S004137474	*Proteobacteria*	*Gammaproteobacteria*	*Xanthomonadales*	*Xanthomonadaceae*	*Dyella*	3	4
S004419336	"Candidatus Saccharibacteria"				"Saccharibacteria_genera_incertae_sedis"	3	4
S004420832	*Actinobacteria*	*Actinobacteria*	*Actinomycetales*			3	4
S001793007T	*Actinobacteria*	*Actinobacteria*	*Actinomycetales*	*Cellulomonadaceae*	*Cellulomonas*	3	5
S002443020T	*Actinobacteria*	*Actinobacteria*	*Actinomycetales*	*Streptomycetaceae*	*Streptomyces*	3	5
S000981736T	*Proteobacteria*	*Alphaproteobacteria*	*Rhizobiales*	*Rhizobiaceae*	*Rhizobium*	26	5
S000666774	*Proteobacteria*	*Alphaproteobacteria*	"Alphaproteobacteria_incertae_sedis"		*Rhizomicrobium*	3	6
S000768423T	*Proteobacteria*	*Gammaproteobacteria*	*Xanthomonadales*	*Xanthomonadaceae*	*Rhodanobacter*	3	6
S000870877T	*Proteobacteria*	*Betaproteobacteria*	*Burkholderiales*	*Burkholderiaceae*	*Burkholderia*	3	6
S000891240T	*Actinobacteria*	*Actinobacteria*	*Actinomycetales*	*Microbacteriaceae*	*Microbacterium*	3	6
S001548584T	*Actinobacteria*	*Actinobacteria*	*Actinomycetales*	*Streptomycetaceae*	*Streptomyces*	3	6
S002075043	*Planctomycetes*	*Planctomycetia*	*Planctomycetales*	*Planctomycetaceae*		3	6
S003558649	*Proteobacteria*	*Gammaproteobacteria*	*Xanthomonadales*	*Xanthomonadaceae*		3	6
S003612727	*Actinobacteria*	*Actinobacteria*	*Actinomycetales*	*Microbacteriaceae*	*Humibacter*	3	6

S003689171	*Proteobacteria*	*Alphaproteobacteria*	*Sphingomonadales*	*Sphingomonadaceae*	*Sphingomonas*	3	6

[a] Cluster in the network shown in Fig. 5.

[b] Count of fields that an OTU enriched with R-10 inoculum.

Table S4. Fungal families responsive to R-10 inoculum in 1 MAT samples.

Phylum	Class	Order	Family	AM Fungi Type	Count [a]
**Decreased with R-10 Inoculum**					
Glomeromycota	Glomeromycetes	Archaeosporales	Archaeosporaceae	Indigenous AM fungi	1
Chytridiomycota	Chytridiomycetes	Chytridiales	Chytridiaceae		1
Basidiomycota	Agaricomycetes	Cantharellales	Clavulinaceae		1
Glomeromycota	Glomeromycetes	Diversisporales	Diversisporaceae	Indigenous AM fungi	1
Basidiomycota	Exobasidiomycetes	Malasseziales	Malasseziaceae		1
"Fungi incertae sedis"	"Zygomycota incertae sedis"	"Mucorales 1"	"Mucorales 1 incertae sedis"		1
Ascomycota	Dothideomycetes	Pleosporales	Pleosporaceae		1
Basidiomycota	Agaricomycetes	Geastrales	Sphaerobolaceae		1
Chytridiomycota	Chytridiomycetes	Spizellomycetales	Spizellomycetaceae		1
Glomeromycota	Glomeromycetes	Diversisporales	Acaulosporaceae	Indigenous AM fungi	2
Basidiomycota	Agaricomycetes	Cantharellales	Ceratobasidiaceae		2
Glomeromycota	Glomeromycetes	Glomerales	Claroideoglomeraceae	Indigenous AM fungi	2
Ascomycota	Leotiomycetes	Helotiales	Helotiales incertae sedis		2
Glomeromycota	Glomeromycetes	Paraglomerales	Paraglomeraceae	Indigenous AM fungi	2
Chytridiomycota	Chytridiomycetes	Rhizophydiales	Terramycetaceae		2
Basidiomycota	Tremellomycetes	Tremellales	Tremellaceae		2
Basidiomycota	Agaricomycetes	Agaricales	Tricholomataceae		2
Glomeromycota	Glomeromycetes	Diversisporales	Gigasporaceae	Indigenous AM fungi	3
Glomeromycota	Glomeromycetes	Glomerales	Glomeraceae	Indigenous AM fungi	3

"Fungi incertae sedis"	"Zygomycota incertae sedis"	Mortierellales	Mortierellaceae		3
**Enriched with R-10 Inoculum**					
Basidiomycota	Agaricomycetes	Sebacinales	Sebacinaceae		3
Glomeromycota	Glomeromycetes	Glomerales	Glomeraceae	R-10 fungus	5

[a] Count of fields that a family responded to R-10 inoculum.

Table S5. Taxonomy of the fungal OTUs responsive to R-10 inoculum in 1 MAT samples.

Code	Phylum	Class	Order	Family	Genus	AM Fungi Type
**Decreased with R-10 Inoculum**						
015_Rhz_AJ510243	Glomeromycota	Glomeromycetes	Glomerales	Glomeraceae	*Rhizophagus*	Indigenous AM fungi
038_Rhz_AB812612	Glomeromycota	Glomeromycetes	Glomerales	Glomeraceae	*Rhizophagus*	Indigenous AM fungi
073_UnG_AB369762	Glomeromycota	Glomeromycetes	Glomerales	Glomeraceae	"Uncultured Glomeraceae"	Indigenous AM fungi
107_UnG_AB369767	Glomeromycota	Glomeromycetes	Glomerales	Glomeraceae	"Uncultured Glomeraceae"	Indigenous AM fungi
108_UnG_AB561107	Glomeromycota	Glomeromycetes	Glomerales	Glomeraceae	"Uncultured Glomeraceae"	Indigenous AM fungi
125_Cla_AB812617	Glomeromycota	Glomeromycetes	Glomerales	Claroideoglomeraceae	*Claroideoglomus*	Indigenous AM fungi
137_Cla_AB665521	Glomeromycota	Glomeromycetes	Glomerales	Claroideoglomeraceae	*Claroideoglomus*	Indigenous AM fungi
139_Cla_LC176567	Glomeromycota	Glomeromycetes	Glomerales	Claroideoglomeraceae	*Claroideoglomus*	Indigenous AM fungi
140_Cla_HQ857084	Glomeromycota	Glomeromycetes	Glomerales	Claroideoglomeraceae	*Claroideoglomus*	Indigenous AM fungi
145_Cla_AY639200	Glomeromycota	Glomeromycetes	Glomerales	Claroideoglomeraceae	*Claroideoglomus*	Indigenous AM fungi
148_Cla_AY639343	Glomeromycota	Glomeromycetes	Glomerales	Claroideoglomeraceae	*Claroideoglomus*	Indigenous AM fungi
150_Cla_JN937518	Glomeromycota	Glomeromycetes	Glomerales	Claroideoglomeraceae	*Claroideoglomus*	Indigenous AM fungi
154_Scu_FN547618	Glomeromycota	Glomeromycetes	Diversisporales	Gigasporaceae	*Scutellospora*	Indigenous AM fungi
158_Gig_AM040348	Glomeromycota	Glomeromycetes	Diversisporales	Gigasporaceae	*Gigaspora*	Indigenous AM fungi
159_Gig_AF378453	Glomeromycota	Glomeromycetes	Diversisporales	Gigasporaceae	*Gigaspora*	Indigenous AM fungi
161_Gig_AF378502	Glomeromycota	Glomeromycetes	Diversisporales	Gigasporaceae	*Gigaspora*	Indigenous AM fungi
162_Gig_FR750177	Glomeromycota	Glomeromycetes	Diversisporales	Gigasporaceae	*Gigaspora*	Indigenous AM fungi
164_Gig_GQ229230	Glomeromycota	Glomeromycetes	Diversisporales	Gigasporaceae	*Gigaspora*	Indigenous AM fungi
184_Aca_JF717598	Glomeromycota	Glomeromycetes	Diversisporales	Acaulosporaceae	*Acaulospora*	Indigenous AM fungi

193_Aca_AB935539	Glomeromycota	Glomeromycetes	Diversisporales	Acaulosporaceae	*Acaulospora*	Indigenous AM fungi
195_Aca_AB369793	Glomeromycota	Glomeromycetes	Diversisporales	Acaulosporaceae	*Acaulospora*	Indigenous AM fungi
219_Par_AB369810	Glomeromycota	Glomeromycetes	Paraglomerales	Paraglomeraceae	*Paraglomus*	Indigenous AM fungi
220_Par_NG_027567	Glomeromycota	Glomeromycetes	Paraglomerales	Paraglomeraceae	*Paraglomus*	Indigenous AM fungi
235_Fun_EU234489_S003836248	Glomeromycota	Glomeromycetes	Glomerales	Glomeraceae	*Funneliformis*	Indigenous AM fungi
290_Gig_FN547583_S003844972	Glomeromycota	Glomeromycetes	Diversisporales	Gigasporaceae	*Gigaspora*	Indigenous AM fungi
291_Gig_FN547596_S003844985	Glomeromycota	Glomeromycetes	Diversisporales	Gigasporaceae	*Gigaspora*	Indigenous AM fungi
296_Scu_FM876834_S003844418	Glomeromycota	Glomeromycetes	Diversisporales	Gigasporaceae	*Scutellospora*	Indigenous AM fungi
327_Aca_AB369790_S002040113	Glomeromycota	Glomeromycetes	Diversisporales	Acaulosporaceae	*Acaulospora*	Indigenous AM fungi
361_Aca_LC191603	Glomeromycota	Glomeromycetes	Diversisporales	Acaulosporaceae	*Acaulospora*	Indigenous AM fungi
366_Scu_LC191606	Glomeromycota	Glomeromycetes	Diversisporales	Gigasporaceae	*Scutellospora*	Indigenous AM fungi
396_Arc_LC191632	Glomeromycota	Glomeromycetes	Archaeosporales	Archaeosporaceae	*Archaeospora*	Indigenous AM fungi
399_Scu_LC191634	Glomeromycota	Glomeromycetes	Diversisporales	Gigasporaceae	*Scutellospora*	Indigenous AM fungi
426_Aca_LC191655	Glomeromycota	Glomeromycetes	Diversisporales	Acaulosporaceae	*Acaulospora*	Indigenous AM fungi
429_Cla_LC191658	Glomeromycota	Glomeromycetes	Glomerales	Claroideoglomeraceae	*Claroideoglomus*	Indigenous AM fungi
S001208702	Ascomycota	Dothideomycetes	Pleosporales	Pleosporaceae	“unclassified_Pleosporaceae”	
S003444217	Basidiomycota	Agaricomycetes	Cantharellales	Ceratobasidiaceae	“unclassified_Ceratobasidiaceae”	
S003812115	Chytridiomycota	Chytridiomycetes	Spizellomycetales	Spizellomycetaceae	*Rhizophlyctis*	
S003820904	Basidiomycota	Agaricomycetes	Geastrales	Sphaerobolaceae	*Sphaerobolus*	
S003822806	Basidiomycota	Agaricomycetes	Cantharellales	Ceratobasidiaceae	“unclassified_Ceratobasidiaceae”	
S003822807	Basidiomycota	Agaricomycetes	Cantharellales	Ceratobasidiaceae	*Thanatephorus*	
S003859109	Basidiomycota	Agaricomycetes	Cantharellales	Clavulinaceae	*Multiclavula*	
S003867652	Basidiomycota	Agaricomycetes	Agaricales	Tricholomataceae	*Mycenella*	

S004039428	Basidiomycota	Agaricomycetes	Auriculariales	Exidiaceae	"unclassified_Exidiaceae"	
S004060842	Ascomycota	Leotiomycetes	Helotiales	"Helotiales incertae sedis"	*Spirosphaera*	
S004098782	"Fungi incertae sedis"	"Zygomycota incertae sedis"	Mortierellales	Mortierellaceae	"unclassified_Mortierellaceae"	
S004258266	"Fungi incertae sedis"	"Zygomycota incertae sedis"	"Mucorales 1"	"Mucorales 1 incertae sedis"	*Umbelopsis*	
S004262133	Basidiomycota	Agaricomycetes	Cantharellales	Ceratobasidiaceae	*Thanatephorus*	
023_Rhz_AB370888	Glomeromycota	Glomeromycetes	Glomerales	Glomeraceae	*Rhizophagus*	Indigenous AM fungi
133_Cla_AB812593	Glomeromycota	Glomeromycetes	Glomerales	Claroideoglomeraceae	*Claroideoglomus*	Indigenous AM fungi
155_Scu_JF816949	Glomeromycota	Glomeromycetes	Diversisporales	Gigasporaceae	*Scutellospora*	Indigenous AM fungi
156_Scu_FR750142	Glomeromycota	Glomeromycetes	Diversisporales	Gigasporaceae	*Scutellospora*	Indigenous AM fungi
287_Scu_HE962462_S004126952	Glomeromycota	Glomeromycetes	Diversisporales	Gigasporaceae	*Scutellospora*	Indigenous AM fungi
S003832081	Basidiomycota	Tremellomycetes	Tremellales	Tremellaceae	*Cryptococcus*	
S003867654	Basidiomycota	Agaricomycetes	Agaricales	Tricholomataceae	*Mycenella*	
S004058177	Chytridiomycota	Chytridiomycetes	Rhizophydiales	Terramycetaceae	*Boothiomyces*	
S004257774	Basidiomycota	Tremellomycetes	Tremellales	Tremellaceae	*Cryptococcus*	
152_Scu_FM876838	Glomeromycota	Glomeromycetes	Diversisporales	Gigasporaceae	*Scutellospora*	Indigenous AM fungi
157_UnG_AB665510	Glomeromycota	Glomeromycetes	Glomerales	Glomeraceae	"Uncultured Glomeraceae"	Indigenous AM fungi
163_Gig_AB665514	Glomeromycota	Glomeromycetes	Diversisporales	Gigasporaceae	*Gigaspora*	Indigenous AM fungi
165_Gig_FN547559	Glomeromycota	Glomeromycetes	Diversisporales	Gigasporaceae	*Gigaspora*	Indigenous AM fungi
**Enriched with R-10 Inoculum**						
007_Rhz_AB369745	Glomeromycota	Glomeromycetes	Glomerales	Glomeraceae	*Rhizophagus*	R-10 fungus
025_Fun_LC014181	Glomeromycota	Glomeromycetes	Glomerales	Glomeraceae	*Funneliformis*	Indigenous AM fungi
026_Fun_AB812585	Glomeromycota	Glomeromycetes	Glomerales	Glomeraceae	*Funneliformis*	Indigenous AM fungi
034_Fun_FN547491	Glomeromycota	Glomeromycetes	Glomerales	Glomeraceae	*Funneliformis*	Indigenous AM fungi

229_Par_AB547188	Glomeromycota	Glomeromycetes	Paraglomerales	Paraglomeraceae	*Paraglomus*	Indigenous AM fungi
S003444313	Basidiomycota	Agaricomycetes	Cantharellales	Ceratobasidiaceae	“unclassified_Ceratobasidiaceae”	
S003876257	Ascomycota	Dothideomycetes	Pleosporales	Pleosporaceae	*Edenia*	
001_Rhz_AB369921	Glomeromycota	Glomeromycetes	Glomerales	Glomeraceae	*Rhizophagus*	R-10 fungus
018_Rhz_JN937265	Glomeromycota	Glomeromycetes	Glomerales	Glomeraceae	*Rhizophagus*	Indigenous AM fungi
230_Rhz_JN937313	Glomeromycota	Glomeromycetes	Glomerales	Glomeraceae	*Rhizophagus*	R-10 fungus
373_Rhz_LC191612	Glomeromycota	Glomeromycetes	Glomerales	Glomeraceae	*Rhizophagus*	R-10 fungus
374_Rhz_LC191613	Glomeromycota	Glomeromycetes	Glomerales	Glomeraceae	*Rhizophagus*	R-10 fungus
006_Rhz_FM865608	Glomeromycota	Glomeromycetes	Glomerales	Glomeraceae	*Rhizophagus*	R-10 fungus
008_Rhz_AB640745	Glomeromycota	Glomeromycetes	Glomerales	Glomeraceae	*Rhizophagus*	R-10 fungus
367_Rhz_LC191607	Glomeromycota	Glomeromycetes	Glomerales	Glomeraceae	*Rhizophagus*	R-10 fungus
S003873577	Basidiomycota	Agaricomycetes	Sebacinales	Sebacinaceae	*Piriformospora*	
231_Rhz_FR750064	Glomeromycota	Glomeromycetes	Glomerales	Glomeraceae	*Rhizophagus*	R-10 fungus
379_Rhz_LC191618	Glomeromycota	Glomeromycetes	Glomerales	Glomeraceae	*Rhizophagus*	R-10 fungus
380_Rhz_LC191619	Glomeromycota	Glomeromycetes	Glomerales	Glomeraceae	*Rhizophagus*	R-10 fungus
388_Rhz_LC191626	Glomeromycota	Glomeromycetes	Glomerales	Glomeraceae	*Rhizophagus*	R-10 fungus
391_Rhz_LC191628	Glomeromycota	Glomeromycetes	Glomerales	Glomeraceae	*Rhizophagus*	R-10 fungus
019_Rhz_AB640747	Glomeromycota	Glomeromycetes	Glomerales	Glomeraceae	*Rhizophagus*	R-10 fungus
392_Rhz_LC191629	Glomeromycota	Glomeromycetes	Glomerales	Glomeraceae	*Rhizophagus*	R-10 fungus

Table S6. Taxonomy of the CDR and CER bacterial OTUs in 1 MAT samples.

RDP Code	Phylum	Class	Order	Family	Genus	Source [a]
**CDR OTUs**						
S000592822T	*Proteobacteria*	*Gammaproteobacteria*	*Xanthomonadales*	*Xanthomonadaceae*	*Rhodanobacter*	Indigenous
S000723697	*Proteobacteria*	*Alphaproteobacteria*	*Rhodospirillales*	*Acetobacteraceae*		Indigenous
S002501429	*Armatimonadetes*	*Armatimonadia*	*Armatimonadales*	*Armatimonadaceae*	"Armatimonas/Armatimonadetes_gp1"	Indigenous
S002756114	*Planctomycetes*	*Planctomycetia*	*Planctomycetales*	*Planctomycetaceae*	*Singulisphaera*	Indigenous
S003229953	*Proteobacteria*	*Alphaproteobacteria*	"Alphaproteobacteria_incertae_sedis"		*Rhizomicrobium*	Indigenous
S000627903T	*Proteobacteria*	*Gammaproteobacteria*	*Xanthomonadales*	*Xanthomonadaceae*	*Dyella*	Indigenous
S000903060T	*Proteobacteria*	*Betaproteobacteria*	*Burkholderiales*	*Burkholderiaceae*	*Burkholderia*	Indigenous
S000903329T	*Proteobacteria*	*Alphaproteobacteria*	*Rhizobiales*	*Phyllobacteriaceae*	*Mesorhizobium*	Indigenous
S000942115T	*Actinobacteria*	*Actinobacteria*	*Actinomycetales*	*Microbacteriaceae*	*Leifsonia*	Indigenous
S002222493T	*Proteobacteria*	*Alphaproteobacteria*	*Rhizobiales*	*Methylobacteriaceae*	*Methylobacterium*	Indigenous
**CER OTUs**						
S000829450	*Proteobacteria*	*Betaproteobacteria*	*Burkholderiales*	"Burkholderiales_incertae_sedis"		Indigenous
S000843269T	*Actinobacteria*	*Actinobacteria*	*Actinomycetales*	*Microbacteriaceae*	*Leifsonia*	Indigenous
S000933778	*Planctomycetes*	*Planctomycetia*	*Planctomycetales*	*Planctomycetaceae*	*Gemmata*	Indigenous
S001019570T	*Proteobacteria*	*Betaproteobacteria*	*Burkholderiales*	*Burkholderiales_incertae_sedis*	*Rubrivivax*	Indigenous
S001148263	*Planctomycetes*	*Planctomycetia*	*Planctomycetales*	*Planctomycetaceae*		Indigenous
S001290680	"Candidatus Saccharibacteria"				"Saccharibacteria_genera_incertae_sedis"	Indigenous
S002226884T	*Proteobacteria*	*Alphaproteobacteria*	*Sphingomonadales*	*Sphingomonadaceae*	*Sphingomonas*	Indigenous
S000571799	*Proteobacteria*	*Alphaproteobacteria*	*Rhizobiales*			Indigenous

S001575978T	*Proteobacteria*	*Alphaproteobacteria*	*Rhizobiales*	*Rhizobiales_incertae_sedis*	*Vasilyevaea*	Indigenous
S000981736T	*Proteobacteria*	*Alphaproteobacteria*	*Rhizobiales*	*Rhizobiaceae*	*Rhizobium*	R-10 inoculum
S001793007T	*Actinobacteria*	*Actinobacteria*	*Actinomycetales*	*Cellulomonadaceae*	*Cellulomonas*	R-10 inoculum
S002443020T	*Actinobacteria*	*Actinobacteria*	*Actinomycetales*	*Streptomycetaceae*	*Streptomyces*	R-10 inoculum
S000666774	*Proteobacteria*	*Alphaproteobacteria*	"Alphaproteobacteria_incertae_sedis"		*Rhizomicrobium*	R-10 inoculum
S000768423T	*Proteobacteria*	*Gammaproteobacteria*	*Xanthomonadales*	*Xanthomonadaceae*	*Rhodanobacter*	R-10 inoculum
S000870877T	*Proteobacteria*	*Betaproteobacteria*	*Burkholderiales*	*Burkholderiaceae*	*Burkholderia*	R-10 inoculum
S000891240T	*Actinobacteria*	*Actinobacteria*	*Actinomycetales*	*Microbacteriaceae*	*Microbacterium*	R-10 inoculum
S001548584T	*Actinobacteria*	*Actinobacteria*	*Actinomycetales*	*Streptomycetaceae*	*Streptomyces*	R-10 inoculum
S002075043	*Planctomycetes*	*Planctomycetia*	*Planctomycetales*	*Planctomycetaceae*		R-10 inoculum
S003558649	*Proteobacteria*	*Gammaproteobacteria*	*Xanthomonadales*	*Xanthomonadaceae*		R-10 inoculum
S003612727	*Actinobacteria*	*Actinobacteria*	*Actinomycetales*	*Microbacteriaceae*	*Humibacter*	R-10 inoculum
S003689171	*Proteobacteria*	*Alphaproteobacteria*	*Sphingomonadales*	*Sphingomonadaceae*	*Sphingomonas*	R-10 inoculum

[a] Shows whether a bacterial OTU is inoculum-associated or indigenous.

Table S7. Taxonomy of the bacterial OTUs clustered in cluster-3 (R-10 cluster) in the network shown in Fig. 5.

RDP Code	Phylum	Class	Order	Family	Genus	Type [a]	Count [b]
S001334010	*Proteobacteria*	*Gammaproteobacteria*	*Xanthomonadales*	*Xanthomonadaceae*	*Arenimonas*	Indigenous	1
S001351455T	*Actinobacteria*	*Actinobacteria*	*Actinomycetales*	*Microbacteriaceae*	*Microbacterium*	Indigenous	1
S001566613	*Proteobacteria*	*Betaproteobacteria*				Indigenous	1
S002902753	*Proteobacteria*	*Alphaproteobacteria*	*Rhizobiales*			Indigenous	1
S004044816	"Candidatus Saccharibacteria"				"Saccharibacteria_genera_incertae_sedis"	Indigenous	1
S002074189	*Proteobacteria*	*Deltaproteobacteria*	*Myxococcales*			Indigenous	1
S004342430	*Planctomycetes*	*Planctomycetia*	*Planctomycetales*	*Planctomycetaceae*	*Singulisphaera*	Indigenous	1
S000620069T	*Bacteroidetes*	*Sphingobacteriia*	*Sphingobacteriales*	*Chitinophagaceae*	*Niastella*	Indigenous	2
S002987346	*Actinobacteria*	*Actinobacteria*	*Gaiellales*	*Gaiellaceae*	*Gaiella*	Indigenous	2
S003317506	"Candidatus Saccharibacteria"				"Saccharibacteria_genera_incertae_sedis"	Indigenous	2
S003689166	*Acidobacteria*	"Acidobacteria_Gp1"				Indigenous	2
S001275023	*Proteobacteria*	*Alphaproteobacteria*	*Sphingomonadales*	*Sphingomonadaceae*	*Sphingomonas*	Indigenous	2
S003808232	*Proteobacteria*	*Alphaproteobacteria*	*Rhodospirillales*	*Rhodospirillaceae*	*Dongia*	Indigenous	2
S004421625	*Verrucomicrobia*	"Subdivision3"			"Subdivision3_genera_incertae_sedis"	Indigenous	2
S000389821	*Planctomycetes*	*Planctomycetia*	*Planctomycetales*	*Planctomycetaceae*	*Gemmata*	Indigenous	3
S000712413T	*Proteobacteria*	*Betaproteobacteria*	*Burkholderiales*	"Burkholderiales_incertae_sedis"	*Aquincola*	Indigenous	3
S000960878	*Proteobacteria*	*Deltaproteobacteria*				Indigenous	3
S000727795T	*Proteobacteria*	*Alphaproteobacteria*	*Caulobacterales*	*Caulobacteraceae*	*Caulobacter*	Indigenous	3
S000598282T	*Firmicutes*	*Bacilli*	*Bacillales*	"Bacillaceae 1"	*Bacillus*	Indigenous	3
S000830559	*Proteobacteria*	*Alphaproteobacteria*	*Sphingomonadales*	*Sphingomonadaceae*		Indigenous	3

S003435725	*Bacteroidetes*	*Sphingobacteriia*	*Sphingobacteriales*	*Chitinophagaceae*		Indigenous	3
S003654598						Indigenous	3
S004342399	*Bacteroidetes*	*Sphingobacteriia*	*Sphingobacteriales*	*Sphingobacteriaceae*	*Mucilaginibacter*	Indigenous	3
S000006614T	*Planctomycetes*	*Planctomycetia*	*Planctomycetales*	*Planctomycetaceae*	*Gemmata*	Indigenous	4
S000018839T	*Verrucomicrobia*	*Opitutae*	*Opitutales*	*Opitutaceae*	*Opitutus*	Indigenous	4
S000326793	*Verrucomicrobia*	*Opitutae*	*Opitutales*	*Opitutaceae*	*Opitutus*	Indigenous	4
S000013092T	*Proteobacteria*	*Gammaproteobacteria*	*Legionellales*	*Legionellaceae*	*Legionella*	Indigenous	4
S000413462T	*Proteobacteria*	*Alphaproteobacteria*	*Sphingomonadales*	*Sphingomonadaceae*	*Sphingopyxis*	Indigenous	4
S000620070T	*Bacteroidetes*	*Sphingobacteriia*	*Sphingobacteriales*	*Chitinophagaceae*	*Niastella*	Indigenous	4
S002905094	*Proteobacteria*	*Alphaproteobacteria*	*Rhizobiales*	*Xanthobacteraceae*	*Pseudolabrys*	Indigenous	4
S002151035T	*Actinobacteria*	*Actinobacteria*	*Actinomycetales*	*Microbacteriaceae*	*Microbacterium*	Indigenous	4
S002510159	*Proteobacteria*	*Deltaproteobacteria*	*Myxococcales*			Indigenous	4
S003640652	*Bacteroidetes*	*Sphingobacteriia*	*Sphingobacteriales*	*Chitinophagaceae*		Indigenous	4
S004091371	*Proteobacteria*	*Alphaproteobacteria*	*Rhodospirillales*	*Rhodospirillaceae*	*Dongia*	Indigenous	4
S000829450	*Proteobacteria*	*Betaproteobacteria*	*Burkholderiales*	"Burkholderiales_incertae_sedis"		Indigenous	5
S000843269T	*Actinobacteria*	*Actinobacteria*	*Actinomycetales*	*Microbacteriaceae*	*Leifsonia*	Indigenous	5
S001019570T	*Proteobacteria*	*Betaproteobacteria*	*Burkholderiales*	"Burkholderiales_incertae_sedis"	*Rubrivivax*	Indigenous	5
S001148263	*Planctomycetes*	*Planctomycetia*	*Planctomycetales*	*Planctomycetaceae*		Indigenous	5
S000933778	*Planctomycetes*	*Planctomycetia*	*Planctomycetales*	*Planctomycetaceae*	*Gemmata*	Indigenous	5
S001290680	"Candidatus Saccharibacteria"				"Saccharibacteria_genera_incertae_sedis"	Indigenous	5
S002226884T	*Proteobacteria*	*Alphaproteobacteria*	*Sphingomonadales*	*Sphingomonadaceae*	*Sphingomonas*	Indigenous	5
S000571799	*Proteobacteria*	*Alphaproteobacteria*	*Rhizobiales*			Indigenous	6
S001575978T	*Proteobacteria*	*Alphaproteobacteria*	*Rhizobiales*	*Rhizobiales_incertae_sedis*	*Vasilyevaea*	Indigenous	6

S000446585T	*Proteobacteria*	*Gammaproteobacteria*	*Xanthomonadales*	*Xanthomonadaceae*	*Fulvimonas*	R-10 inoculum	1
S001151982T	*Actinobacteria*	*Actinobacteria*	*Actinomycetales*	*Pseudonocardiaceae*	*Actinokineospora*	R-10 inoculum	1
S004196347	*Proteobacteria*	*Betaproteobacteria*	*Burkholderiales*	*Burkholderiaceae*	*Burkholderia*	R-10 inoculum	1
S002291301T	*Proteobacteria*	*Alphaproteobacteria*	*Rhizobiales*	*Bradyrhizobiaceae*	*Nitrobacter*	R-10 inoculum	2
S000651638T	*Proteobacteria*	*Betaproteobacteria*	*Burkholderiales*	*Comamonadaceae*	*Schlegelella*	R-10 inoculum	2
S000013901T	*Proteobacteria*	*Alphaproteobacteria*	*Rhizobiales*	*Hyphomicrobiaceae*	*Hyphomicrobium*	R-10 inoculum	3
S002762975	*Verrucomicrobia*	*Opitutae*	*Opitutales*	*Opitutaceae*	*Opitutus*	R-10 inoculum	3
S004416914	"Candidatus Saccharibacteria"				"Saccharibacteria_genera_incertae_sedis"	R-10 inoculum	3
S000429494T	*Actinobacteria*	*Actinobacteria*	*Actinomycetales*	*Mycobacteriaceae*	*Mycobacterium*	R-10 inoculum	4
S002508026	*Chloroflexi*					R-10 inoculum	4
S004137474	*Proteobacteria*	*Gammaproteobacteria*	*Xanthomonadales*	*Xanthomonadaceae*	*Dyella*	R-10 inoculum	4
S004420832	*Actinobacteria*	*Actinobacteria*	*Actinomycetales*			R-10 inoculum	4
S004419336	"Candidatus Saccharibacteria"				"Saccharibacteria_genera_incertae_sedis"	R-10 inoculum	4
S002443020T	*Actinobacteria*	*Actinobacteria*	*Actinomycetales*	*Streptomycetaceae*	*Streptomyces*	R-10 inoculum	5
S001793007T	*Actinobacteria*	*Actinobacteria*	*Actinomycetales*	*Cellulomonadaceae*	*Cellulomonas*	R-10 inoculum	5
S000768423T	*Proteobacteria*	*Gammaproteobacteria*	*Xanthomonadales*	*Xanthomonadaceae*	*Rhodanobacter*	R-10 inoculum	6
S000666774	*Proteobacteria*	*Alphaproteobacteria*	"Alphaproteobacteria_incertae_sedis"		*Rhizomicrobium*	R-10 inoculum	6
S000870877T	*Proteobacteria*	*Betaproteobacteria*	*Burkholderiales*	*Burkholderiaceae*	*Burkholderia*	R-10 inoculum	6
S000891240T	*Actinobacteria*	*Actinobacteria*	*Actinomycetales*	*Microbacteriaceae*	*Microbacterium*	R-10 inoculum	6
S001548584T	*Actinobacteria*	*Actinobacteria*	*Actinomycetales*	*Streptomycetaceae*	*Streptomyces*	R-10 inoculum	6
S002075043	*Planctomycetes*	*Planctomycetia*	*Planctomycetales*	*Planctomycetaceae*		R-10 inoculum	6
S003558649	*Proteobacteria*	*Gammaproteobacteria*	*Xanthomonadales*	*Xanthomonadaceae*		R-10 inoculum	6
S003612727	*Actinobacteria*	*Actinobacteria*	*Actinomycetales*	*Microbacteriaceae*	*Humibacter*	R-10 inoculum	6

S003689171	*Proteobacteria*	*Alphaproteobacteria*	*Sphingomonadales*	*Sphingomonadaceae*	*Sphingomonas*	R-10 inoculum	6

[a] Shows whether a bacterial OTU is inoculum-associated or indigenous.

[b] Count of fields that a bacterial OTU enriched with R-10 inoculum.

Table S8. Taxonomy of the indigenous bacterial OTUs clustered in cluster-26 in the network shown in Fig. 5 and negatively interacted with R-10 fungus.

RDP Code	Phylum	Class	Order	Family	Genus	Count [a]	Connection [b]
S000115472	*Proteobacteria*	*Betaproteobacteria*	*Burkholderiales*				FALSE
S000840745	*Chloroflexi*	*Ktedonobacteria*	*Ktedonobacterales*				FALSE
S000425929T	*Proteobacteria*	*Betaproteobacteria*	*Burkholderiales*	*Burkholderiaceae*	*Ralstonia*	3	FALSE
S000438365T	*Proteobacteria*	*Alphaproteobacteria*	*Rhizobiales*	*Bradyrhizobiaceae*	*Bradyrhizobium*	2	FALSE
S000818908							FALSE
S001556544	*Bacteroidetes*	*Sphingobacteriia*	*Sphingobacteriales*	*Chitinophagaceae*			FALSE
S001610945	*Proteobacteria*	*Betaproteobacteria*					FALSE
S004052019	*Proteobacteria*	*Betaproteobacteria*	*Burkholderiales*				FALSE
S000391359T	*Proteobacteria*	*Betaproteobacteria*	*Burkholderiales*	*Burkholderiaceae*	*Cupriavidus*		FALSE
S002221828T	*Bacteroidetes*	*Sphingobacteriia*	*Sphingobacteriales*	*Sphingobacteriaceae*	*Mucilaginibacter*	1	FALSE
S001243670T	*Proteobacteria*	*Gammaproteobacteria*	*Pseudomonadales*	*Pseudomonadaceae*	*Pseudomonas*		TRUE
S001226270	*Proteobacteria*	*Alphaproteobacteria*					TRUE
S003435738	*Bacteroidetes*	*Sphingobacteriia*	*Sphingobacteriales*	*Chitinophagaceae*			TRUE
S000389775T	*Actinobacteria*	*Actinobacteria*	*Actinomycetales*	*Micrococcaceae*	*Arthrobacter*	2	TRUE
S000840854	*Armatimonadetes*	*Armatimonadia*	*Armatimonadales*	*Armatimonadaceae*	"Armatimonas/Armatimonadetes_gp1"	1	TRUE

[a] Count of fields that a bacterial OTU decreased with R-10 inoculum.

[b] Shows whether a bacterial OTU was directly connected an indigenous AM fungus of cluster-26.

SUPPLEMENTARY FIGURES

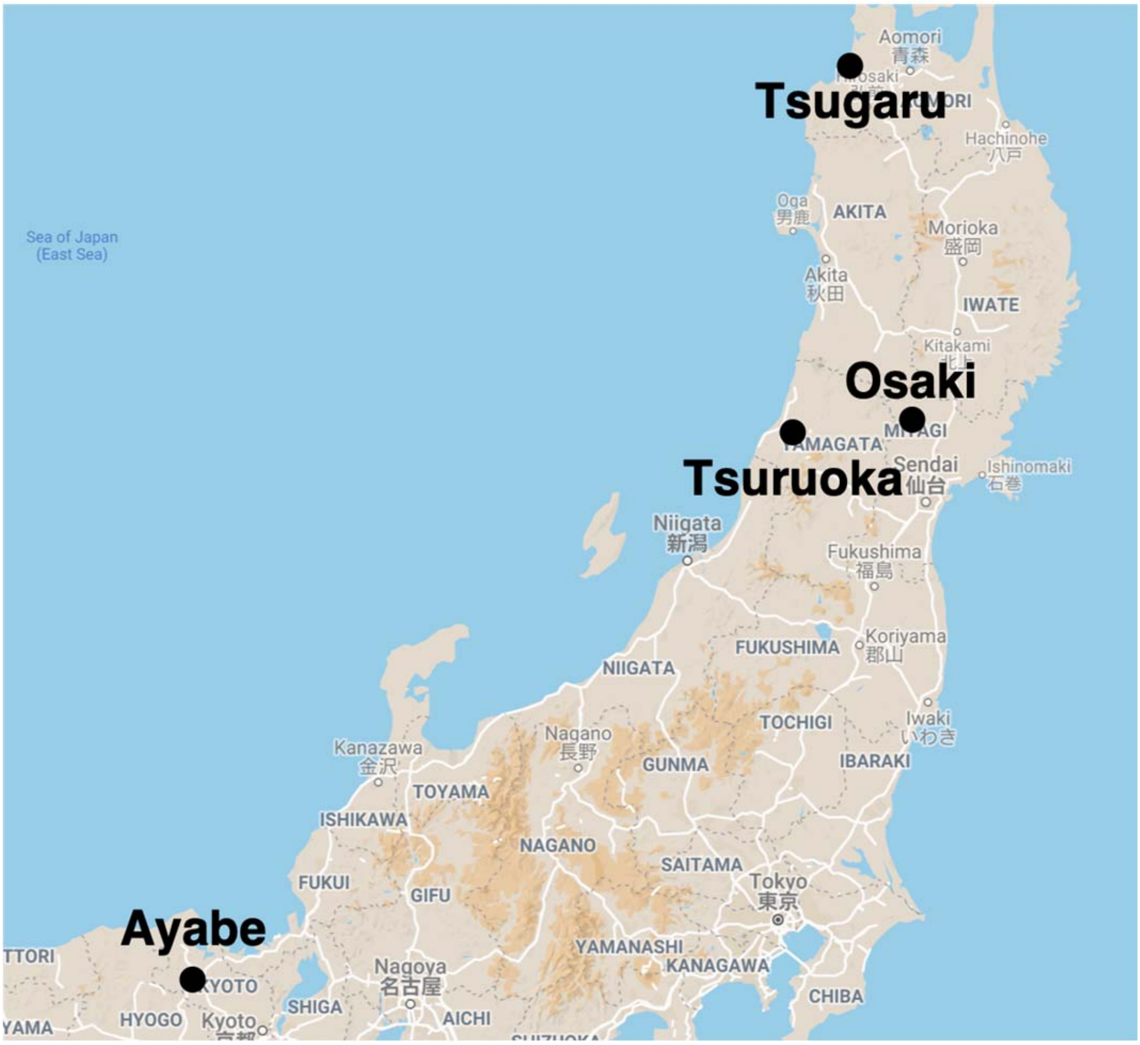

Fig. S1. Map depicting the field locations.

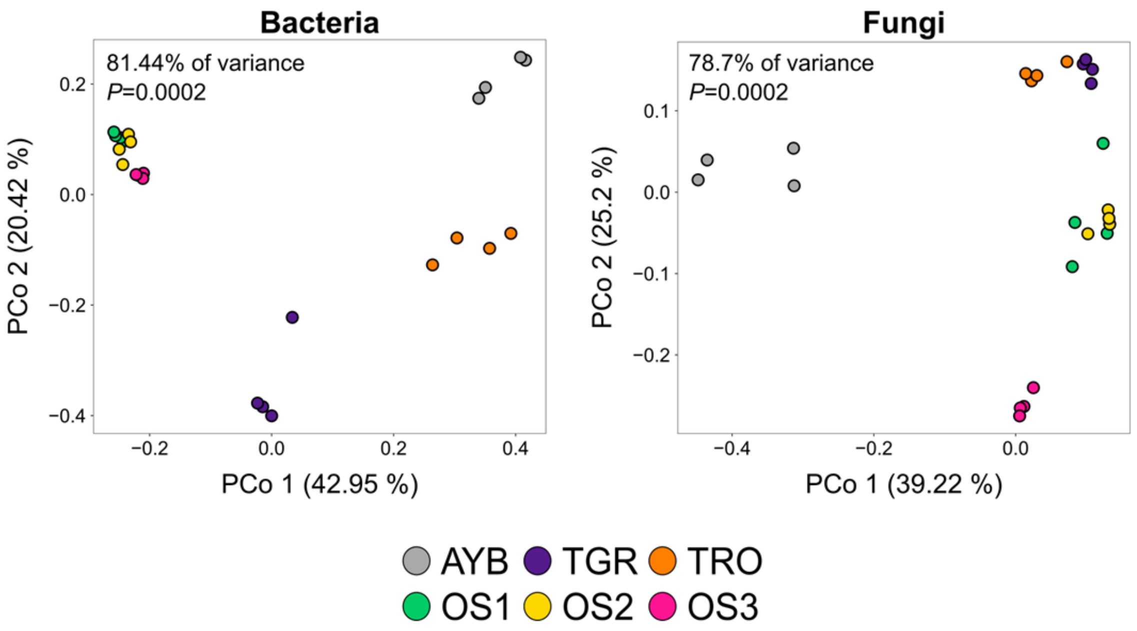

Fig. S2. Unconstrained principal coordinates analysis (PCoA) on Bray-Curtis dissimilarities to show the variation in the bacterial and fungal community structures in bulk soil samples. Variation explained (%) by each axis is given in parenthesis. According to pairwise PERMANOVA, bacterial community structure of the fields was statistically different from each other (P=0.032 for all the comparisons). For the fungal community, we found that OS1 and OS2 fields were not different (P=0.399) but all other comparisons were statistically significant (P=0.034). The P-values for pairwise PERMANOVA were corrected for multiple testing by using Benjamini-Hochberg method.

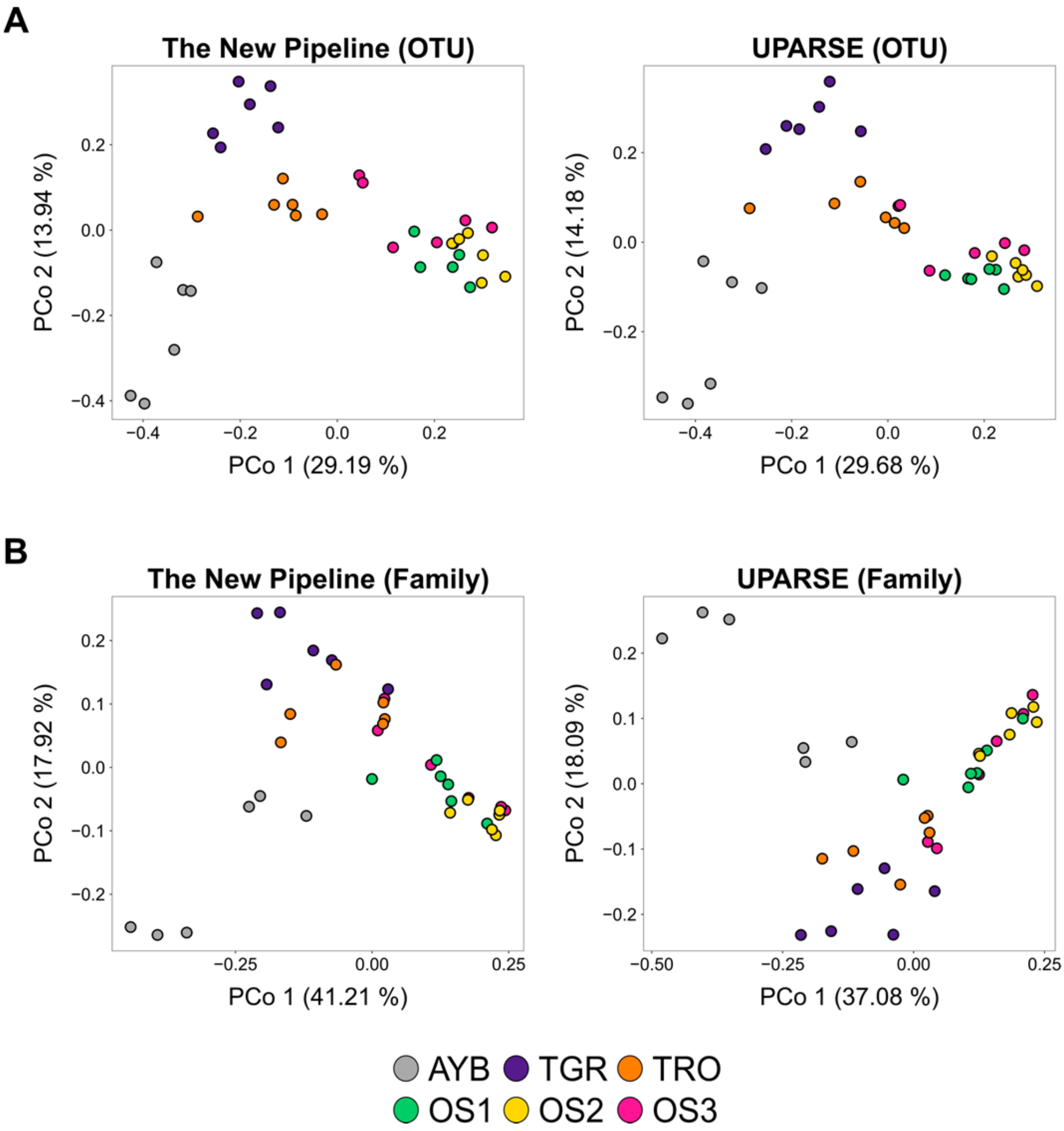

Fig. S3. Unconstrained PCoAs on Bray-Curtis dissimilarities to compare the new pipeline with UPARSE at **(A)** OTU and **(B)** family level. Variation explained (%) by each axis is given in parenthesis. The patterns of the ordinations compared by using Procrustes analysis, which yielded very low errors for both of the comparisons (M^2=0.067 and M^2=0.045 at OTU and family level, respectively).

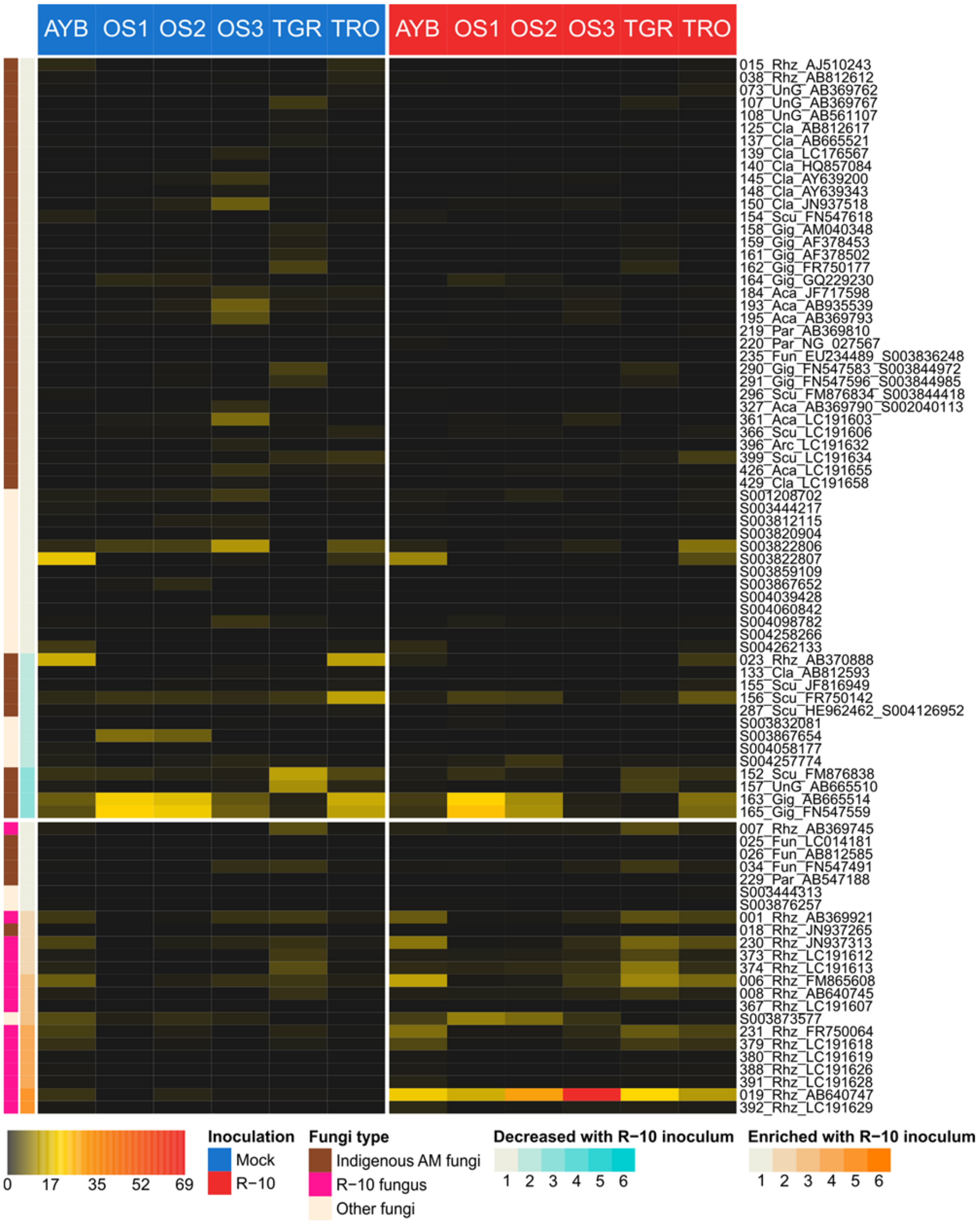

Fig. S4. Heatmap showing the average relative abundance (%) of the fungal OTUs responsive to R-10 inoculum in 1 MAT samples. The average values were calculated by combining samples from different P fertilizer levels (n=12 for AYB, OS3, TGR, and TRO; n=8 for OS1 and OS2). The OTUs decreased or enriched with R-10 inoculum ordered by the number of the fields that they were responsive to R-10 inoculum (indicated with turquoise and orange colors at the left of the heatmap). The type of the fungi was also depicted (R-10 fungus, indigenous AM fungi, and other fungi).

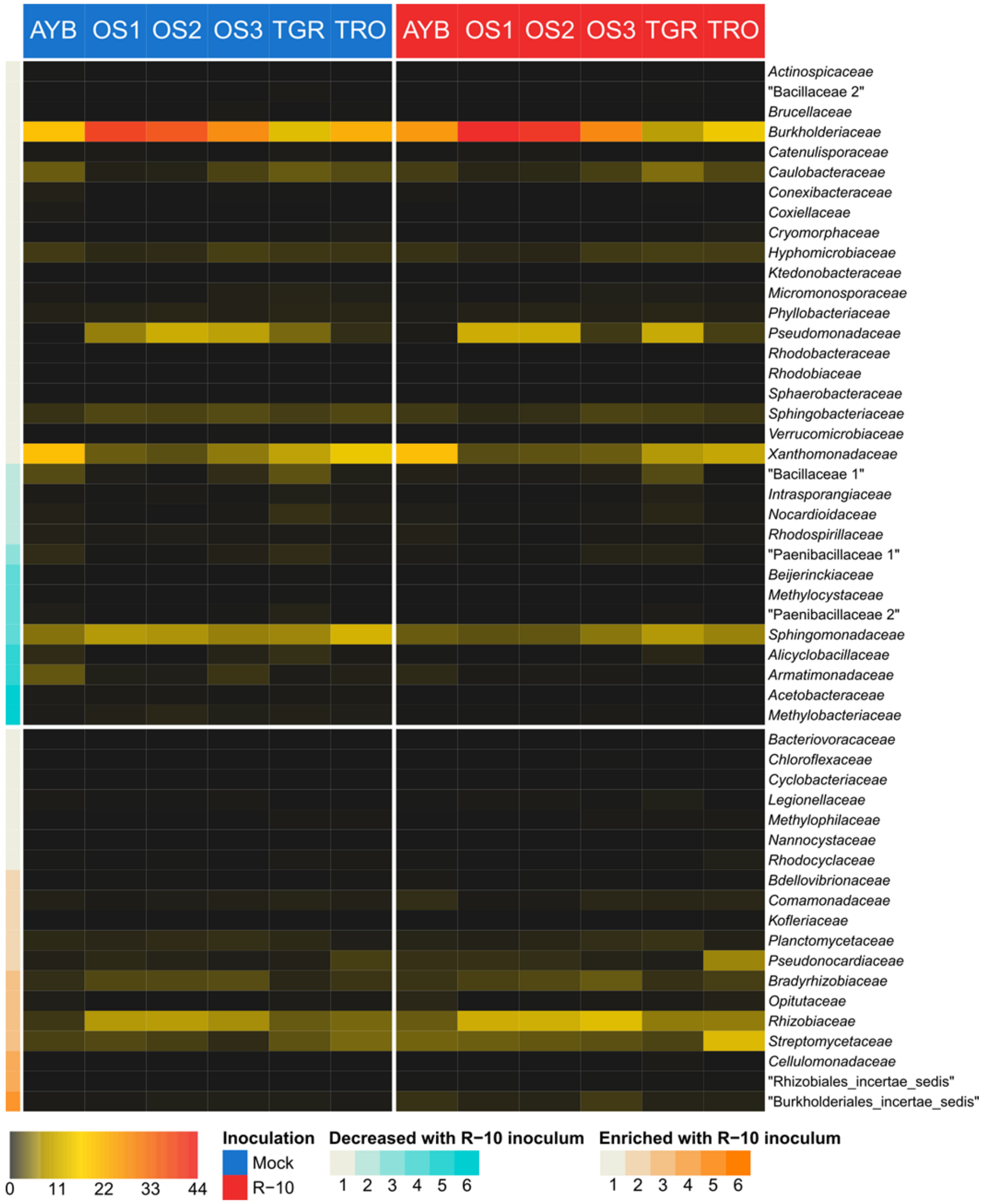

Fig. S5. Heatmap showing the average relative abundance (%) of the bacterial families responsive to R-10 inoculum in 1 MAT samples. The average values were calculated by combining samples from different P fertilizer levels (n=12 for AYB, OS3, TGR, and TRO; n=8 for OS1 and OS2). The families ordered by the number of the fields that they were decreased or enriched with R-10 inoculum (indicated with turquoise and orange colors at the left of the heatmap).

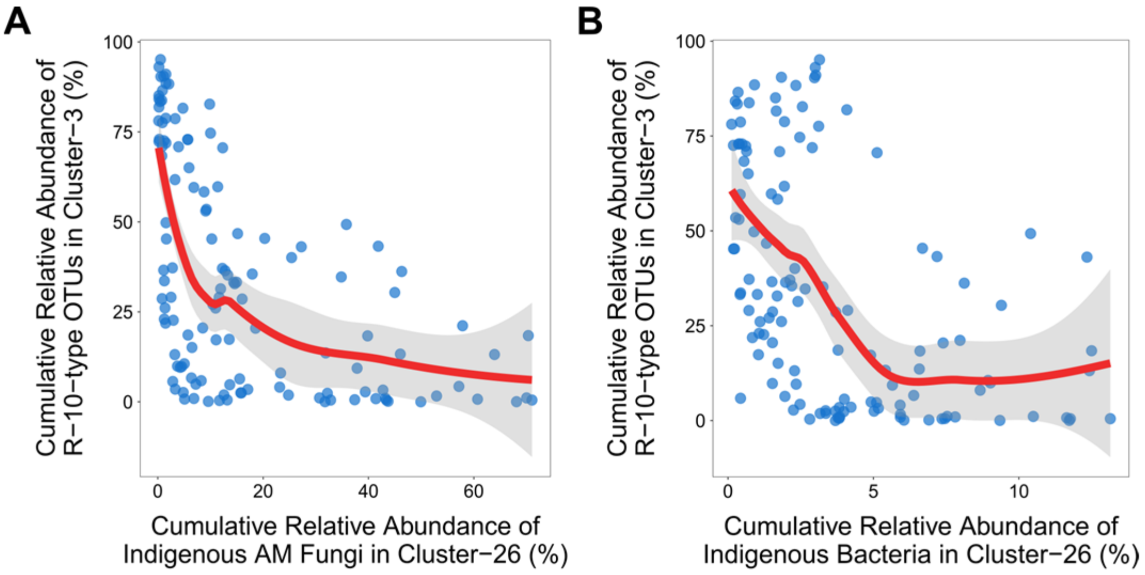

Fig. S6. Interactions between R-10 fungus and indigenous microbes. **(A)** The negative interaction between cluster-26 (indigenous AM fungi) and -3 (R-10 fungus). **(B)** The negative interaction between cluster-26 (indigenous bacteria) and -3 (R-10 fungus).

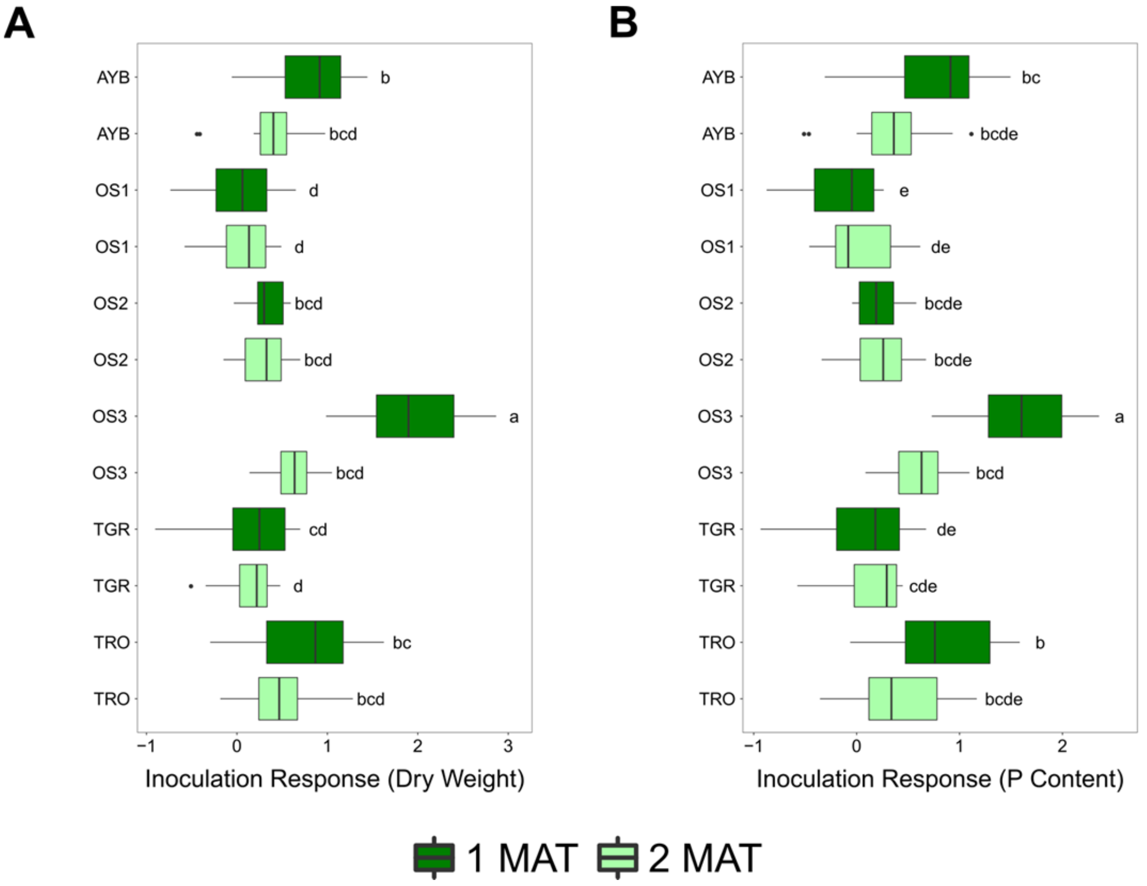

Fig. S7. Effect of R-10 inoculation on the **(A)** dry weight and **(B)** P content of Welsh onion plants. The metric is the log response ratio of R-10 inoculation to mock inoculation, which is positive for a beneficial effect and negative for a detrimental effect. The vertical bars within boxes represent median. The medians were calculated by combining samples from different P fertilizer levels (n=12 for AYB, OS3, TGR, and TRO; n=8 for OS1 and OS2). Letters (a-e) at the right of each whisker indicate statistically significant difference at P<0.05 (one-way ANOVA and Tukey's HSD).
